# Supplementary material for: Unravelling subclonal heterogeneity and aggressive disease states in TNBC through single-cell RNA-seq
Source: Nat Commun. 2018 Sep 4;9:3588. doi: 10.1038/s41467-018-06052-0 (PMC6123496; doi:10.1038/s41467-018-06052-0)
Supplement: Supplementary file 1 — Supplementary Information [file 41467_2018_6052_MOESM1_ESM.pdf]

Unravelling sub-clonal heterogeneity and aggressive disease states in TNBC through single-cell RNA-seq

Mihriban Karaayvaz *[1], Simona Cristea *[2,3,4], Shawn M. Gillespie[1,5], Anoop Patel[6], Ravindra Mylvaganam[1,5], Christina C. Luo[1,5], Michelle C. Specht[7], Bradley E. Bernstein[1,5,8], Franziska Michor †[2,3,4,8,9,10], and Leif W. Ellisen †[1]

[1]Center for Cancer Research, Massachusetts General Hospital, and Harvard Medical School, Boston, MA, 02114, USA.
[2]Department of Biostatistics and Computational Biology, Dana-Farber Cancer Institute, Boston, MA, 02115, USA.
[3]Department of Biostatistics, Harvard T. H. Chan School of Public Health, Boston, MA, 02215, USA.
[4]Department of Stem Cell and Regenerative Biology, Harvard University, Cambridge, MA, 02138, USA.
[5]Department of Pathology, Massachusetts General Hospital, and Harvard Medical School, Boston, MA, 02114, USA.
[6]Department of Neurosurgery, Massachusetts General Hospital, and Harvard Medical School, Boston, MA, 02114, USA.
[7]Department of Surgical Oncology, Massachusetts General Hospital, Boston, MA, 02114, USA.
[8]The Broad Institute of Harvard and MIT, Cambridge, MA, 02139, USA.
[9]Center for Cancer Evolution, Dana-Farber Cancer Institute, Boston, MA 02115, USA.
[10]The Ludwig Center at Harvard, Boston, MA, 02215, USA.

Contents

Supplementary Figures 2

Supplementary Tables 20

Supplementary Methods 25

*equal contribution
†corresponding author

Supplementary Figures

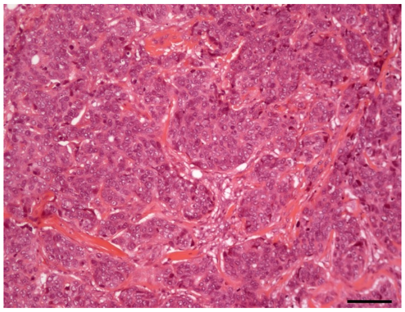

(a) PT039

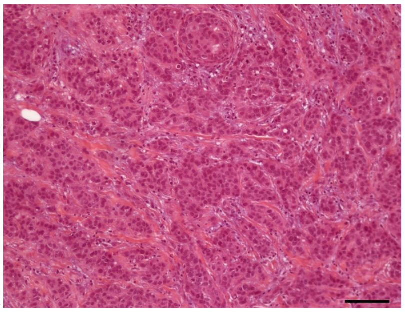

(b) PT058

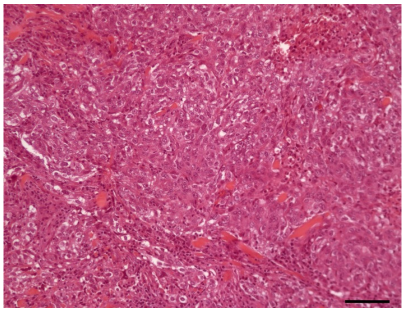

(c) PT081

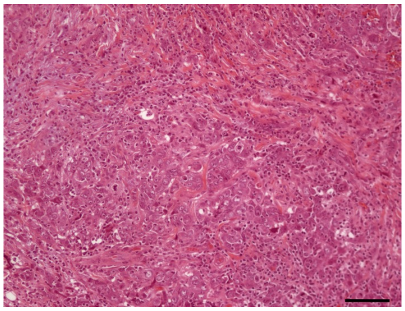

(d) PT084

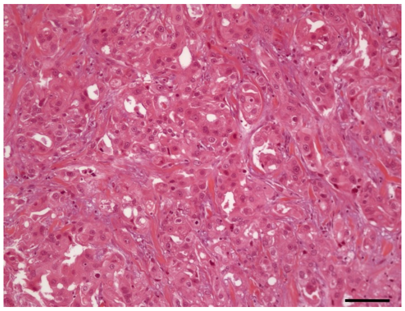

(e) PT089

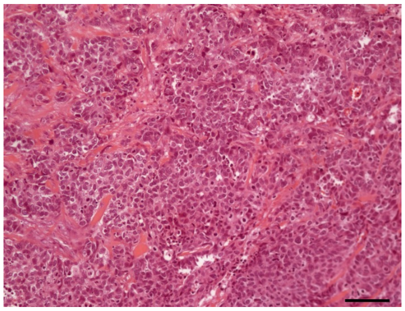

(f) PT126

Supplementary Fig. 1: Histology of the six primary TNBC patients used in this study, showing characteristic cytonuclear morphology. Scale bar represents 100 um.

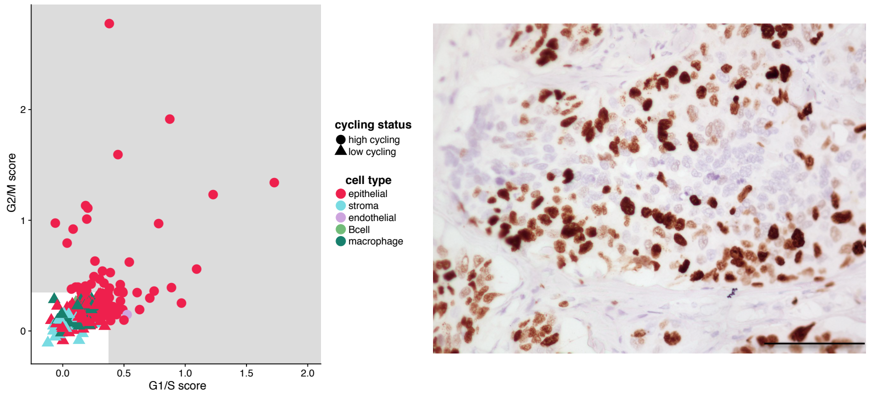

(a) PT039

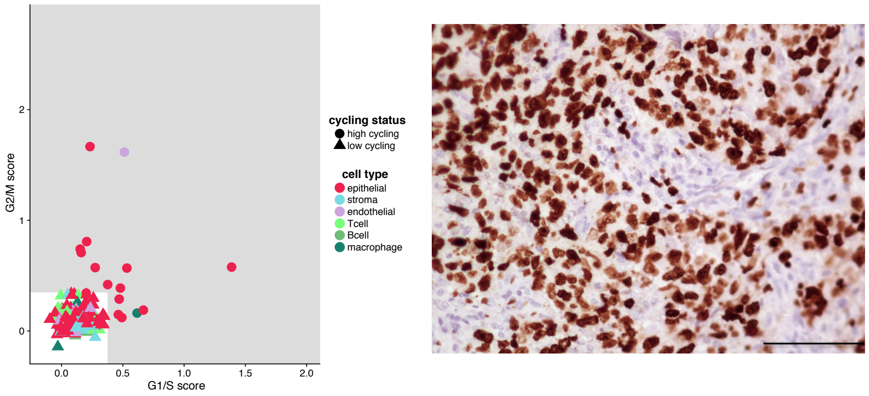

(b) PT084

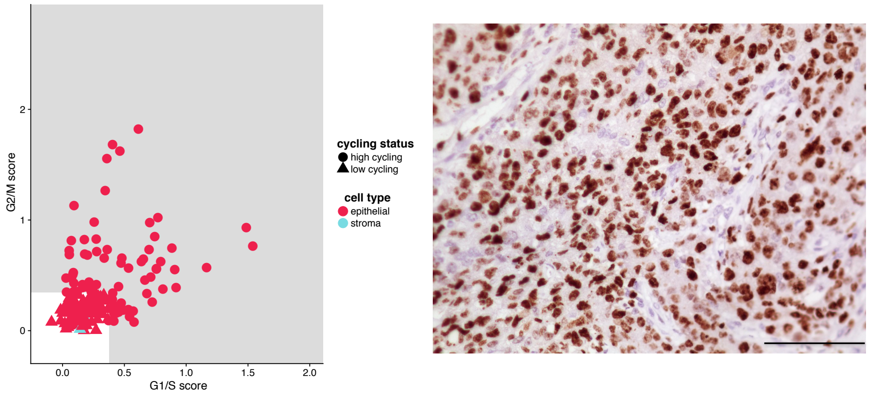

(c) PT081

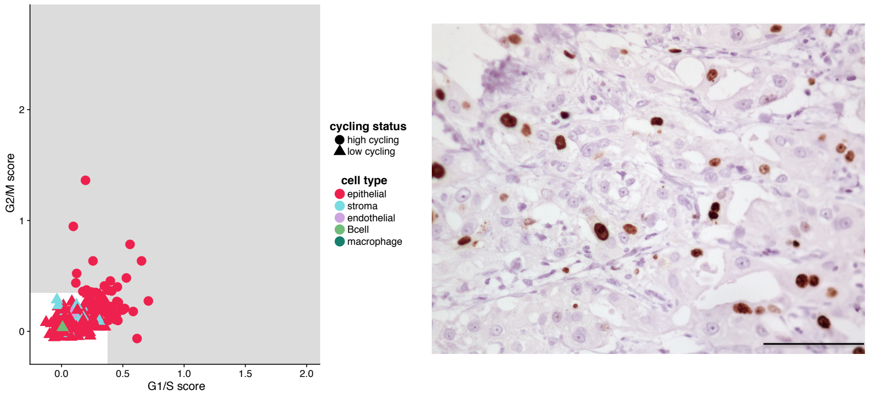

(d) PT089

Supplementary Fig. 2: Proliferation characteristics for four of the six TNBC patients, depicted as either the inferred cycling status of the single cells (left) or Ki67 staining of the tumor (right). Scale bar represents 50 um.

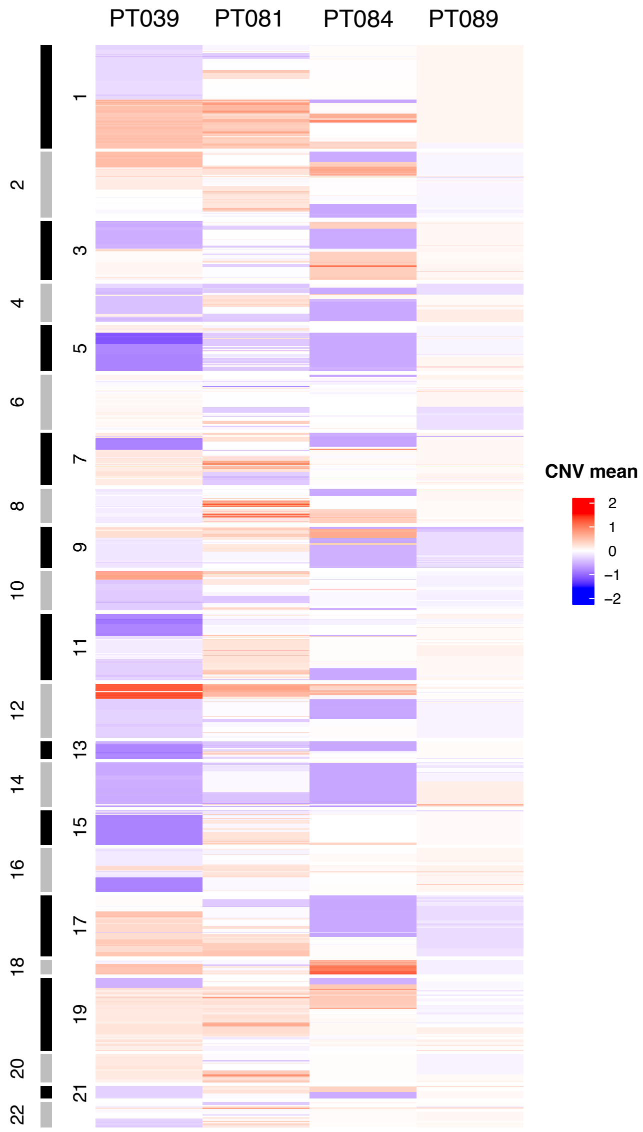

Supplementary Fig. 3: Exome CNV mean gene segments data on all 22, 311 profiled genes.

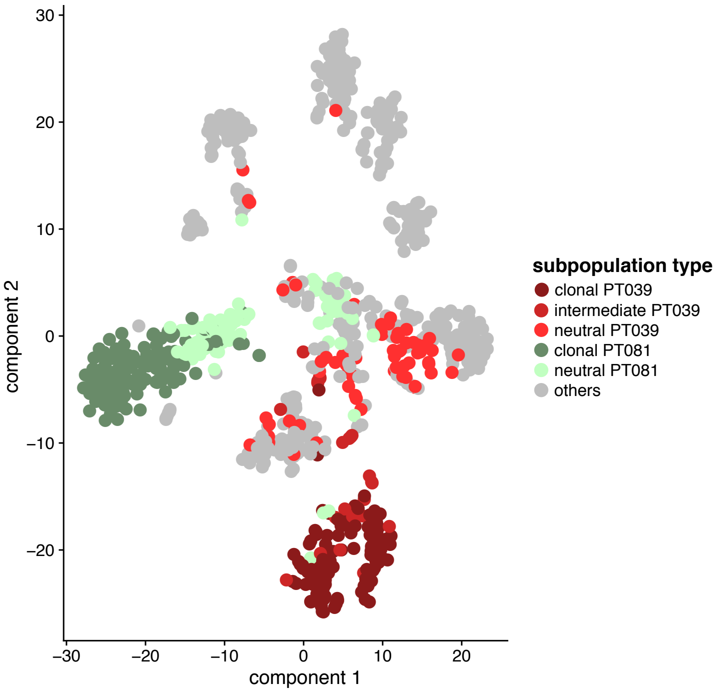

Supplementary Fig. 4: Clonal epithelial subpopulations of patients PT039 and PT081, shown on the t-SNE plot of epithelial cells. The three different epithelial subpopulations for PT039 were the ones determined by the three main branches of the patient-specific hierarchical clustering in Figure 2D in the main text. The two different epithelial subpopulations for PT081 were the ones determined by the two main branches of the hierarchical clustering.

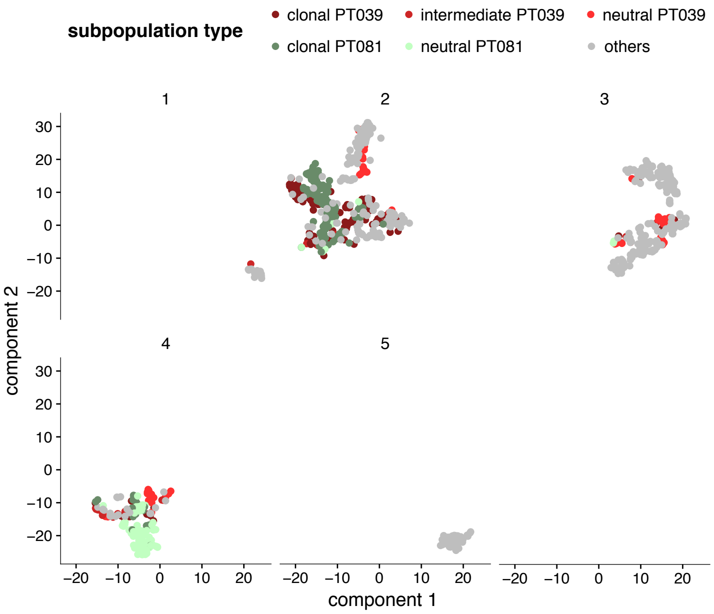

Supplementary Fig. 5: Clonal epithelial subpopulations of patients PT039 and PT081, shown separately for each of the five epithelial clusters identified. The three different epithelial subpopulations for PT039 were the ones determined by the three main branches of the patient-specific hierarchical clustering in Figure 2D in the main text. The two different epithelial subpopulations for PT081 were the ones determined by the two main branches of the hierarchical clustering.

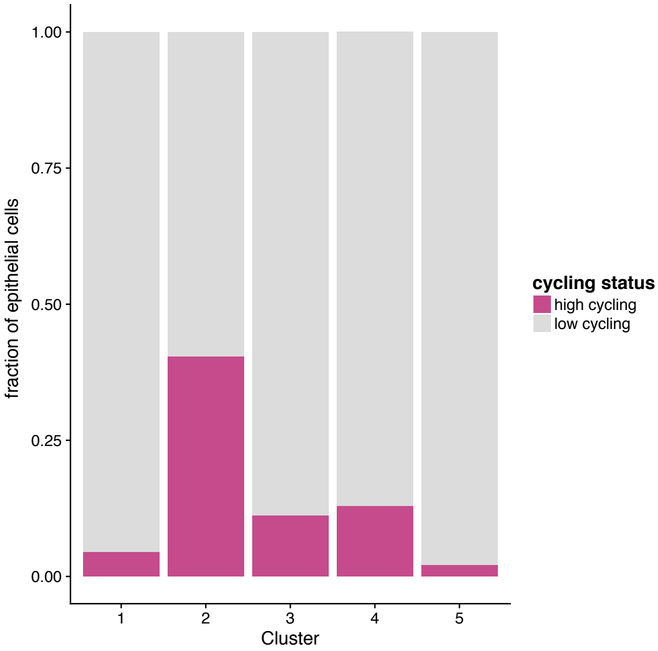

Supplementary Fig. 6: Fraction of high cycling epithelial cells for each of the five epithelial clusters.

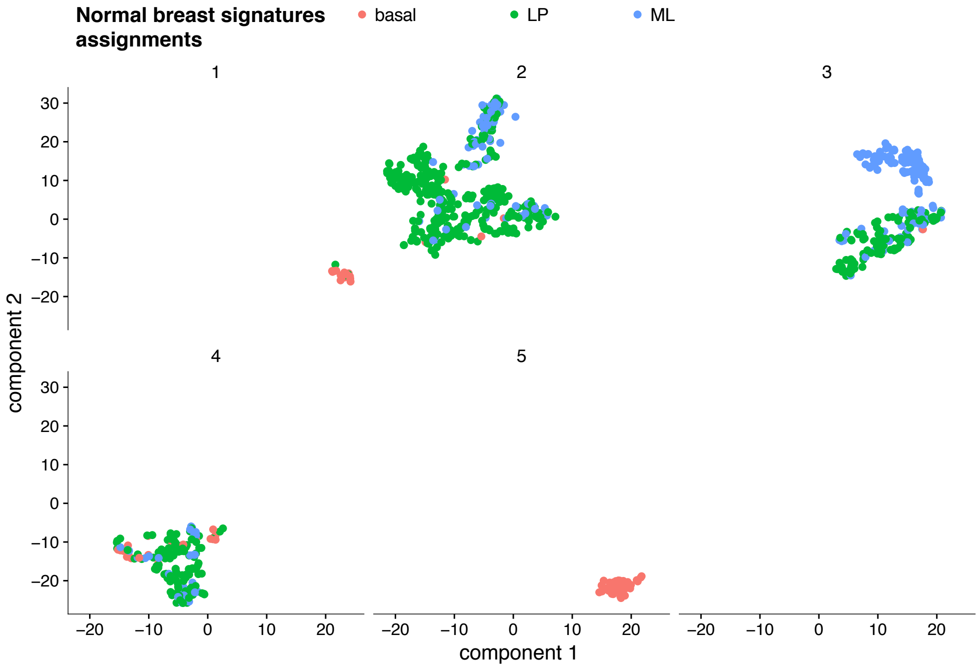

Supplementary Fig. 7: Cluster assignments of the 868 epithelial cells to the normal breast signatures (Lim et al., 2009a). Each cell was assigned to a single signature, depending on the difference between its average expression of the upregulated genes characterizing the signature, and the average expression of the downregulated genes.

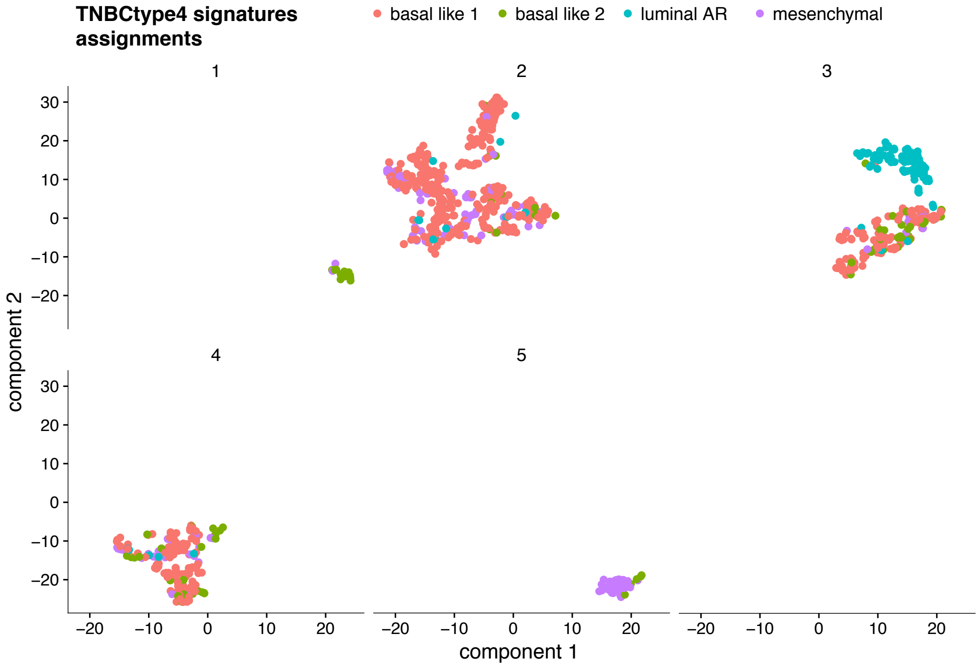

Supplementary Fig. 8: Cluster assignments of the 868 epithelial cells to the TNBCtype4 signatures (Lehmann et al., 2016). Each cell was assigned to a single signature, depending on the difference between its average expression of the upregulated genes characterizing the signature, and the average expression of the downregulated genes.

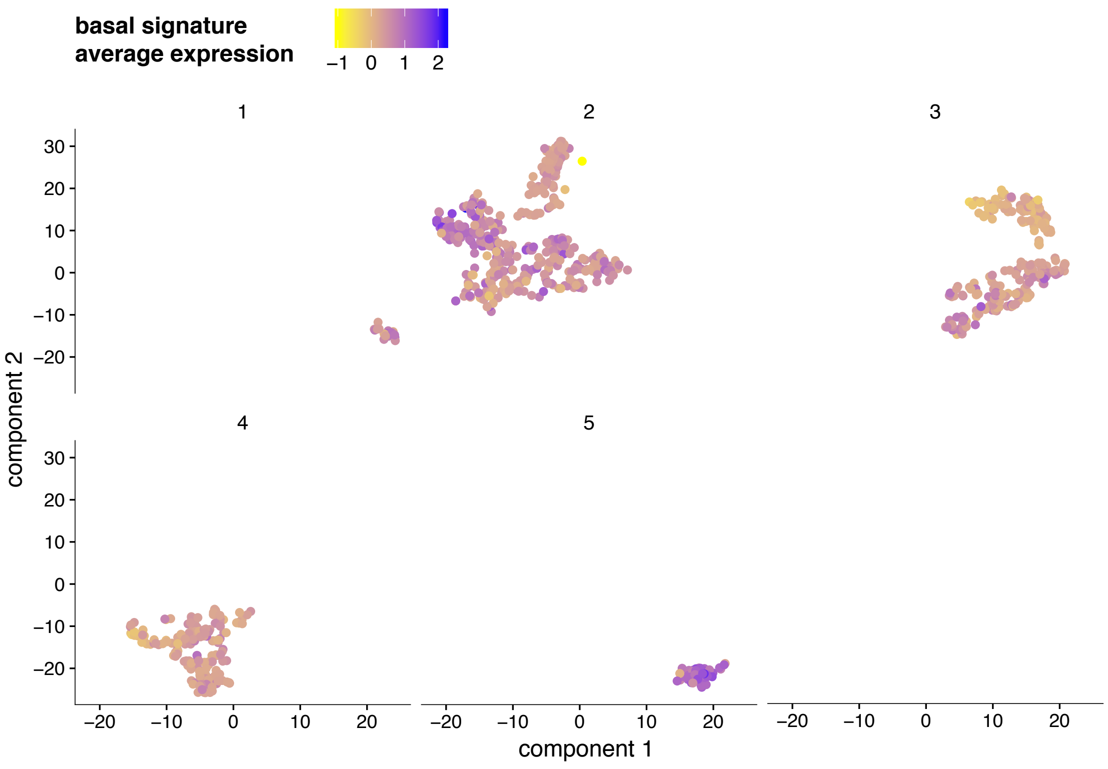

(a) Average expression per cluster.

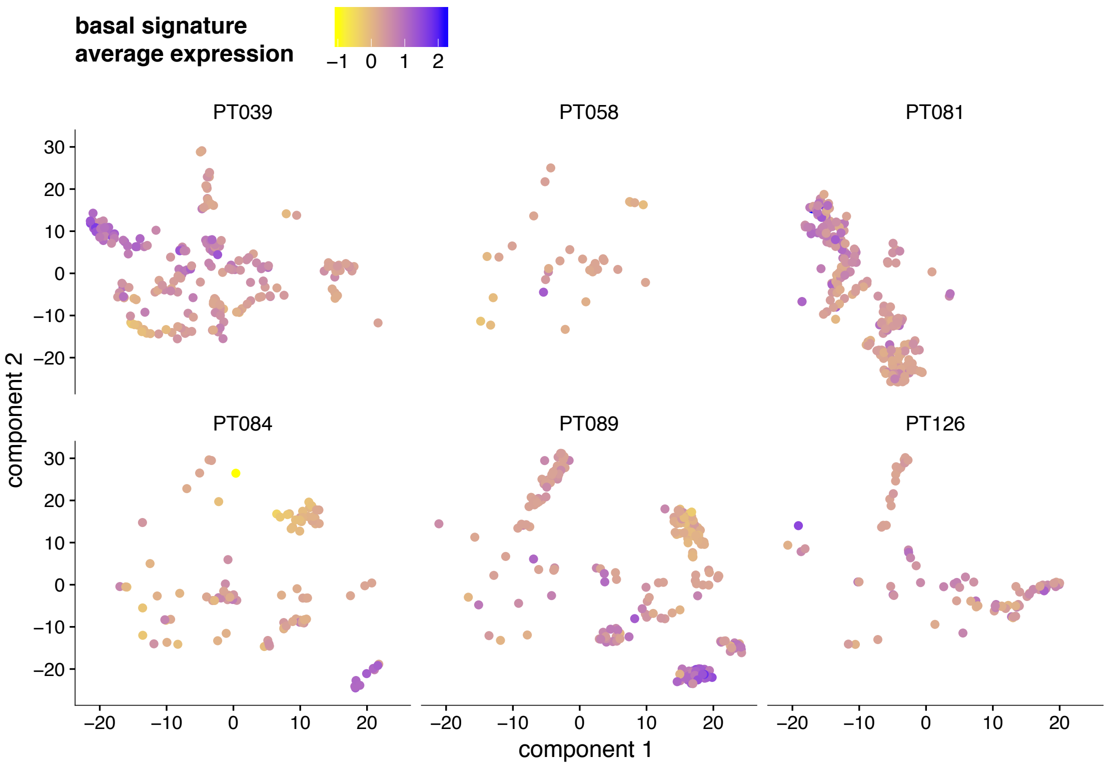

(b) Average expression per patient.

Supplementary Fig. 9: Average expression of the 868 epithelial cells under the Intrinsic Basal signature (Sørlie et al., 2001).

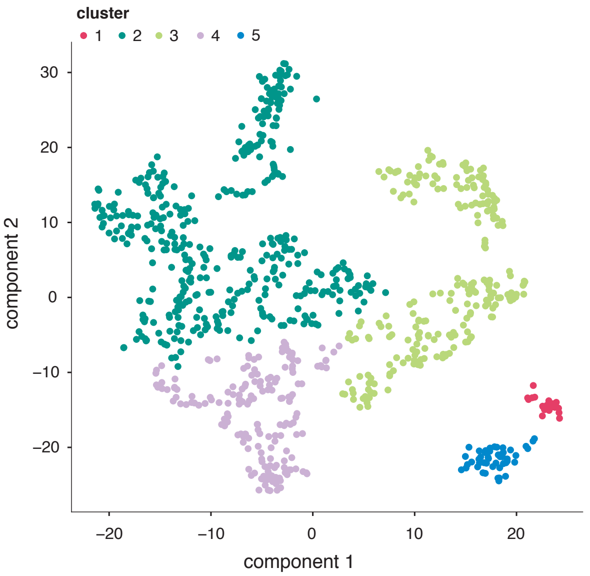

(a) t-SNE plot of epithelial cells, showing five distinct clusters.

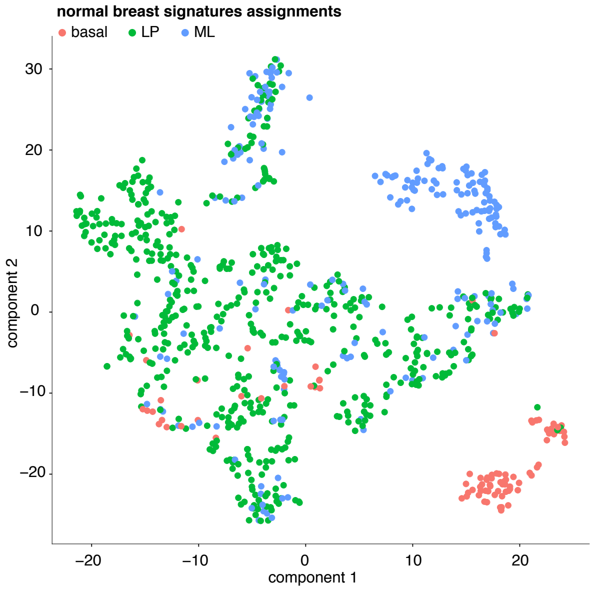

(b) t-SNE plot of epithelial cells, colored by their assignment to the normal signatures.

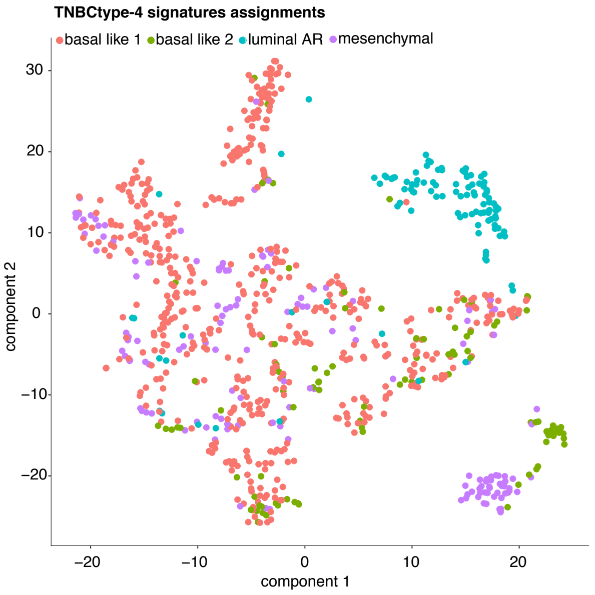

(c) t-SNE plot of epithelial cells, colored by their assignment to the TNBCtype4 signatures.

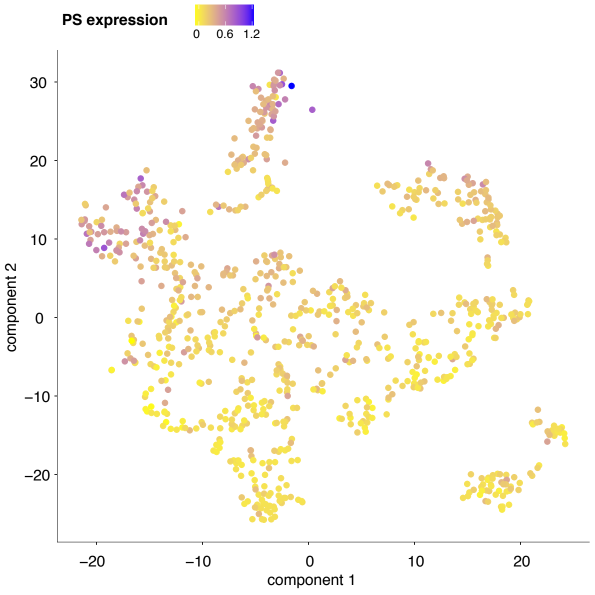

(d) t-SNE plot of epithelial cells, colored by their expression under the 70-gene prognosis signature (PS).

Supplementary Fig. 10: t-SNE plot from Fig. 3a in the main text (a), with cells analyzed for signatures described in the manuscript, including the normal signatures (Lim et al., 2009a) (b), the TNBCtype4 signatures (Lehmann et al., 2016) (c), and the 70-gene prognosis signature (Van't Veer et al., 2002a,b) (d and Fig. 4 in the main text).

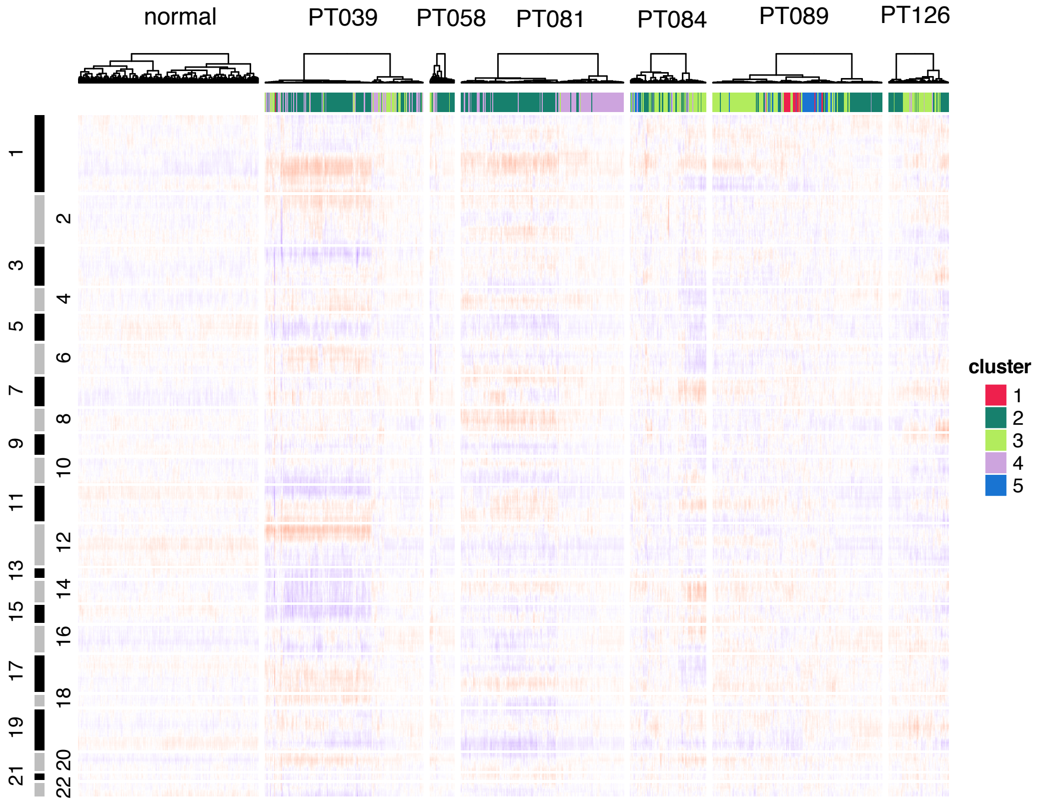

Supplementary Fig. 11: Copy number profiles inferred from single cell gene expression data for the 868 epithelial cells, including cluster assignment.

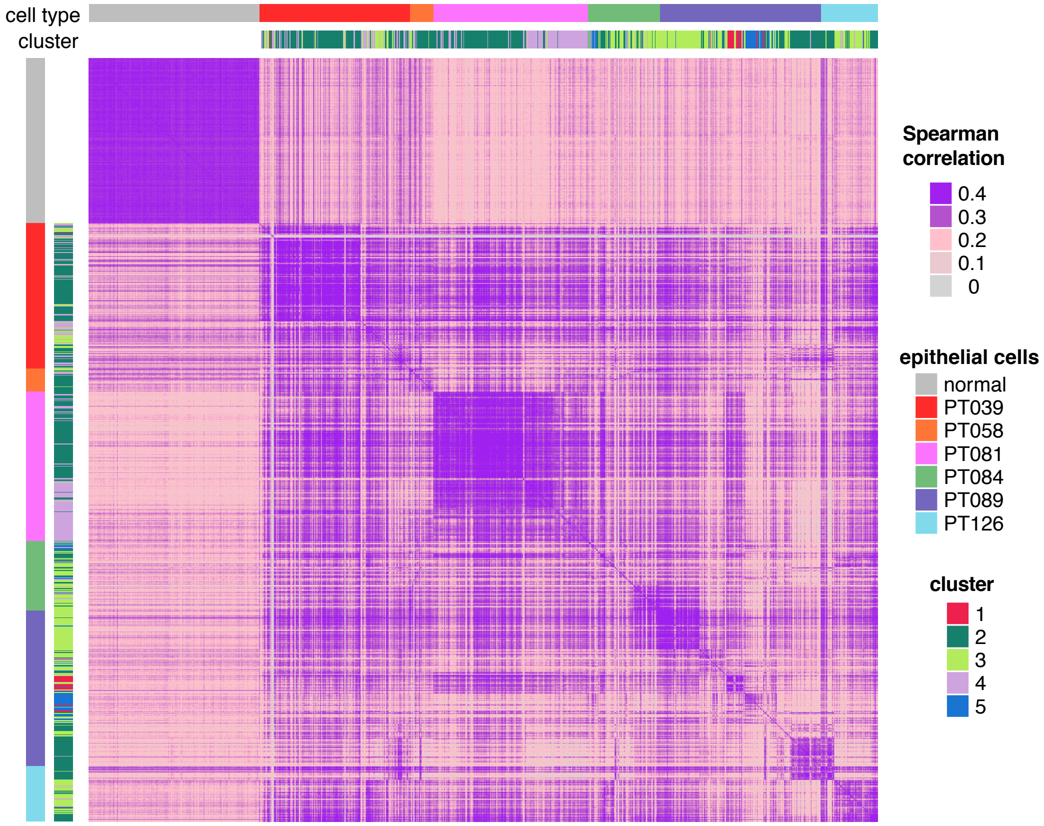

Supplementary Fig. 12: Expression correlation map of the 868 epithelial cells, including cluster assignment.

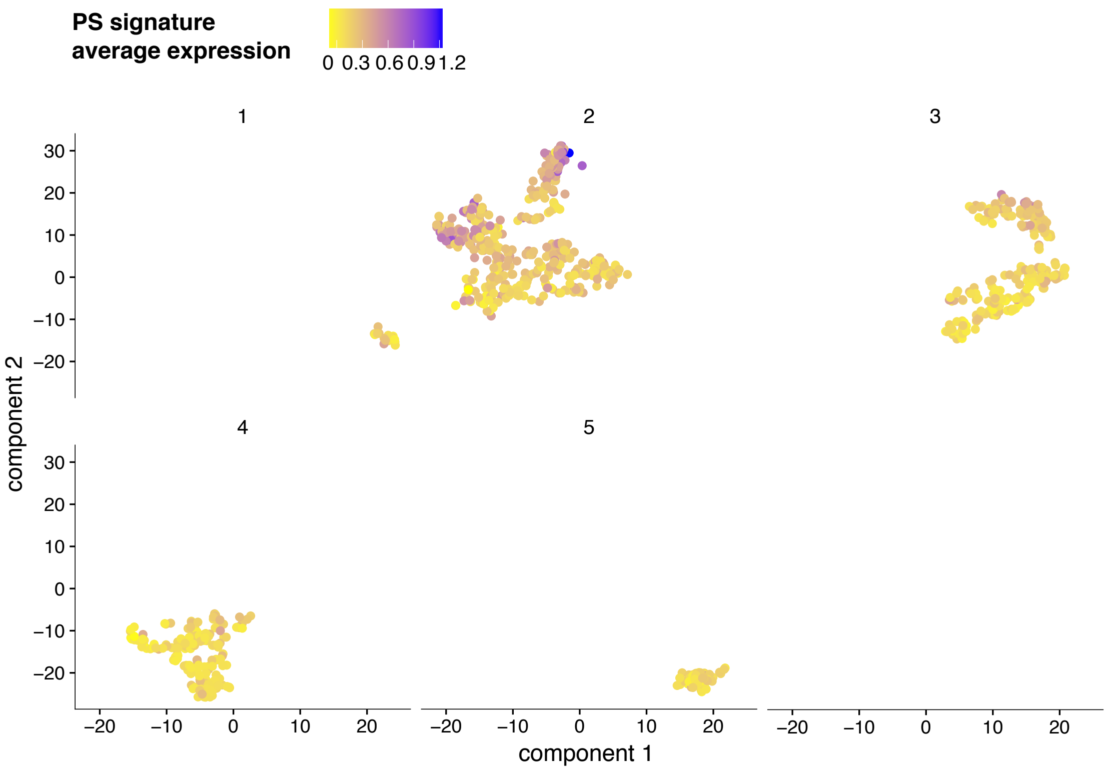

(a) Average expression per cluster.

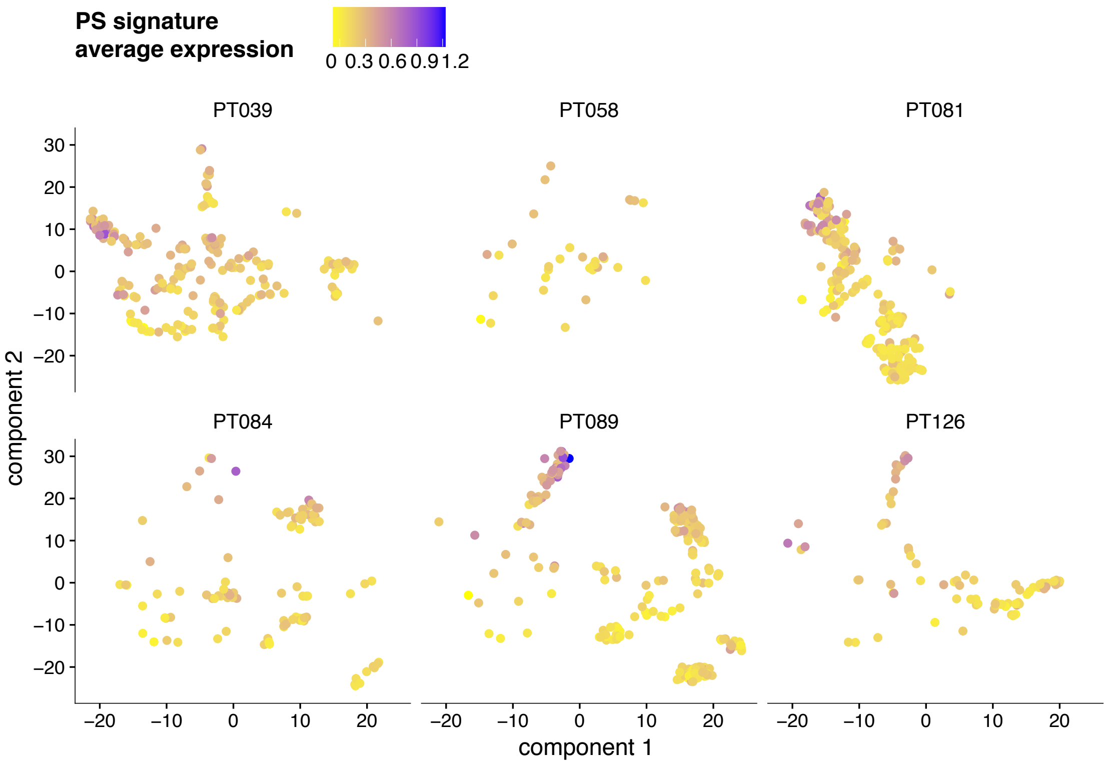

(b) Average expression per patient.

Supplementary Fig. 13: Average expression of the 868 epithelial cells under the 70-gene prognostic signature (PS) (Van't Veer et al., 2002a,b).

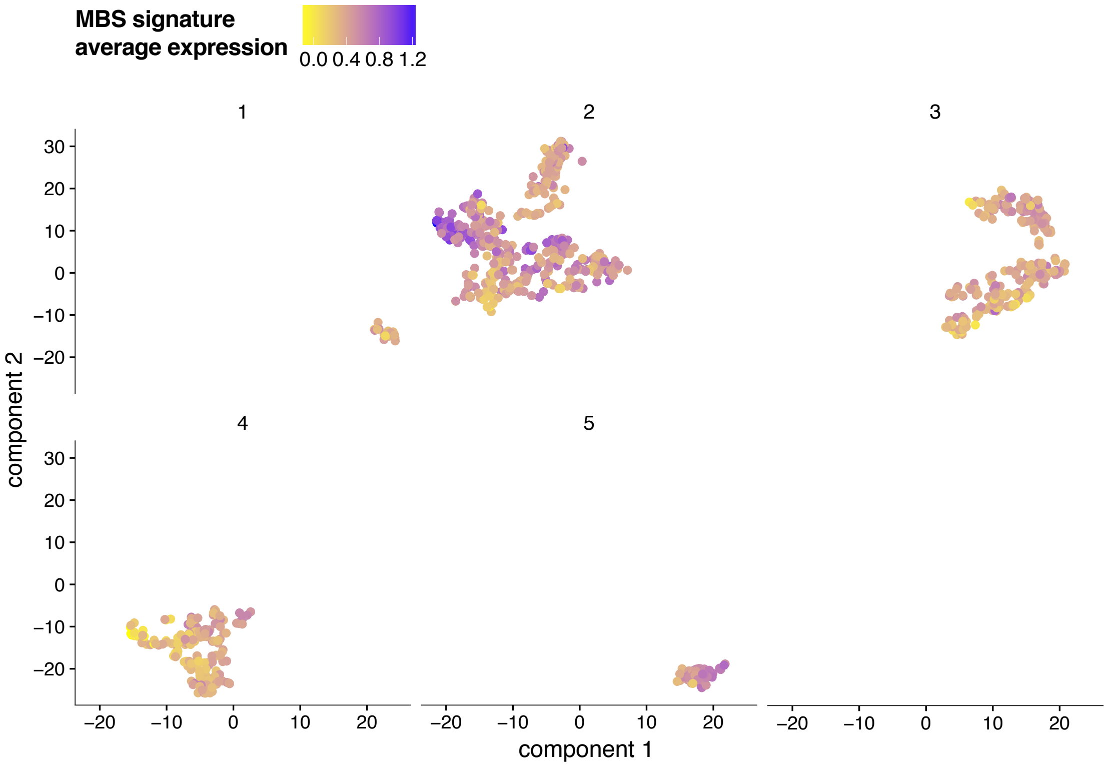

(a) Average expression per cluster.

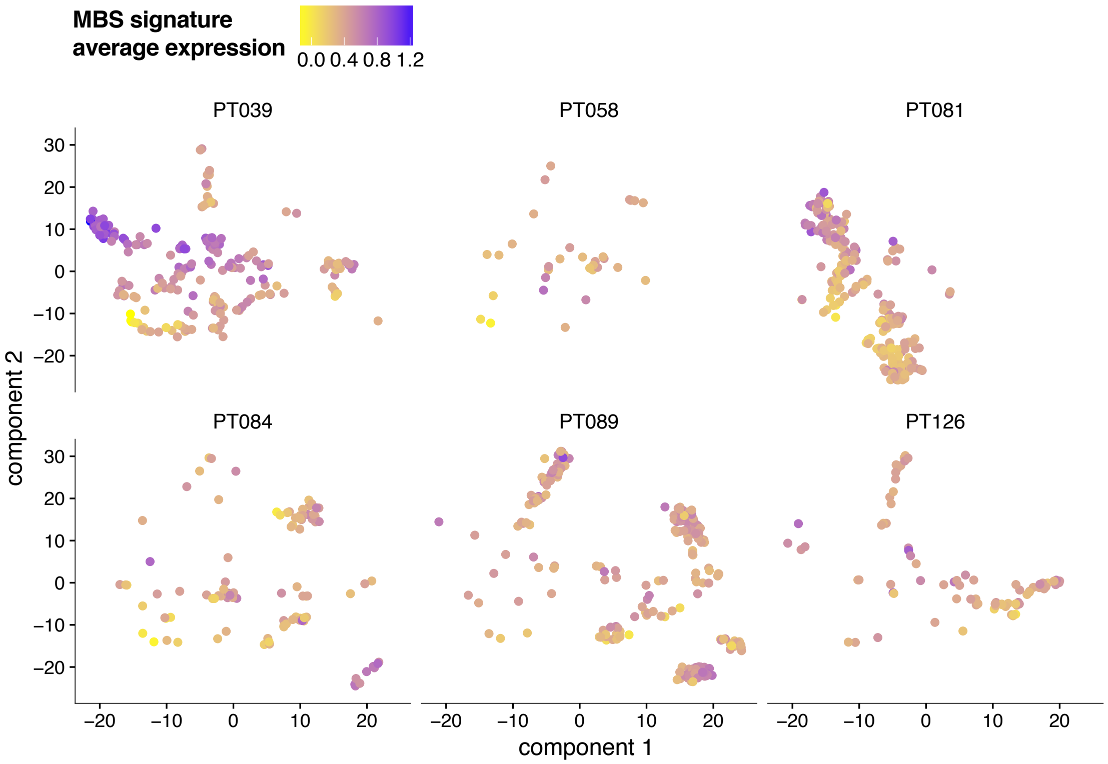

(b) Average expression per patient.

Supplementary Fig. 14: Average expression of the 868 epithelial cells under the 49-gene metastatic burden signature (MBS) (Lawson et al., 2015).

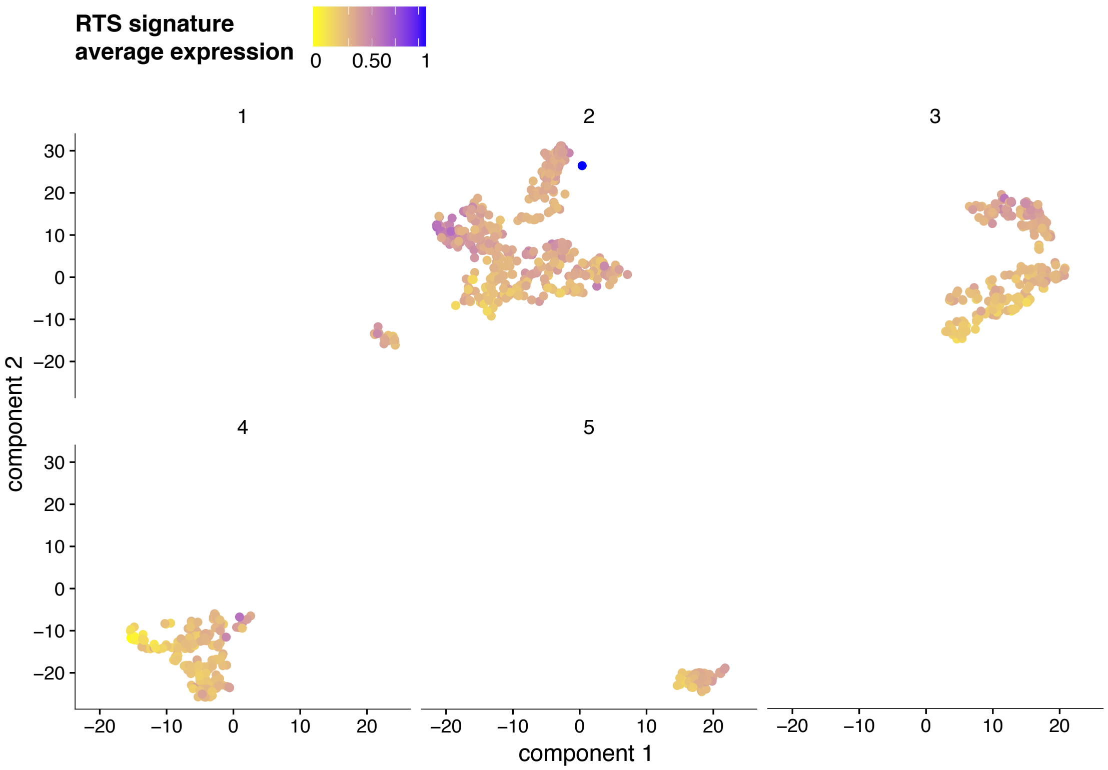

(a) Average expression per cluster.

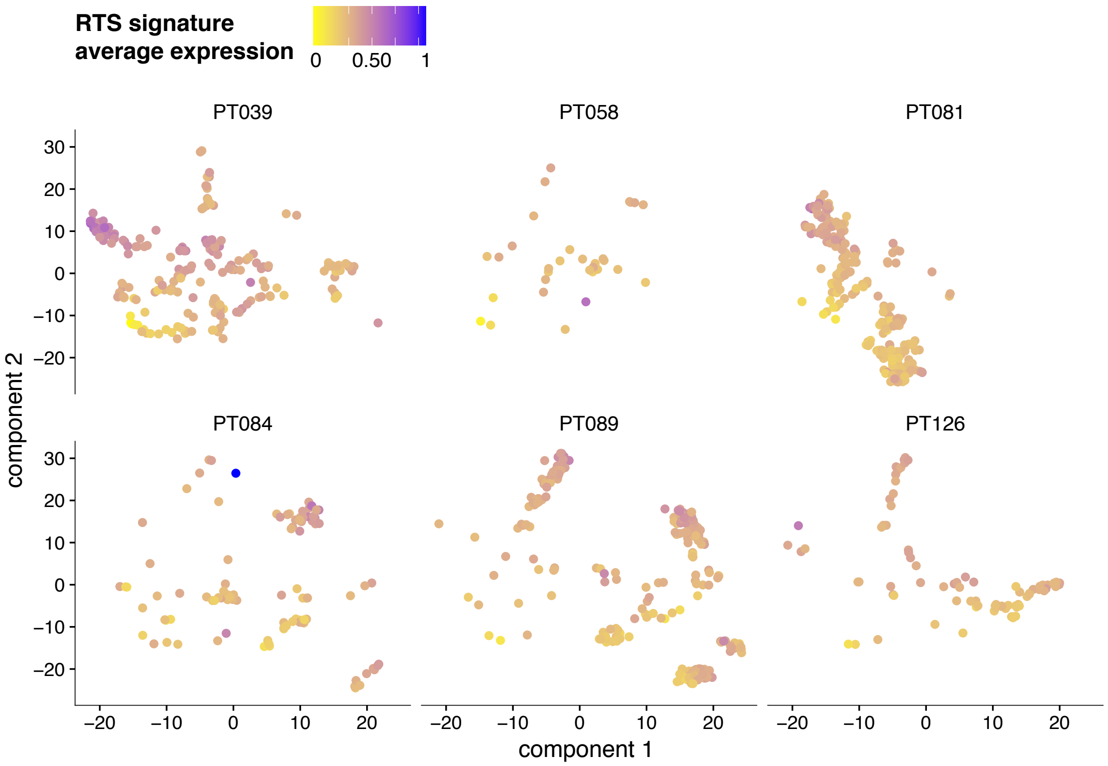

(b) Average expression per patient.

Supplementary Fig. 15: Average expression of the 868 epithelial cells under the 354-gene residual tumor signature (RTS) (Balko et al., 2012).

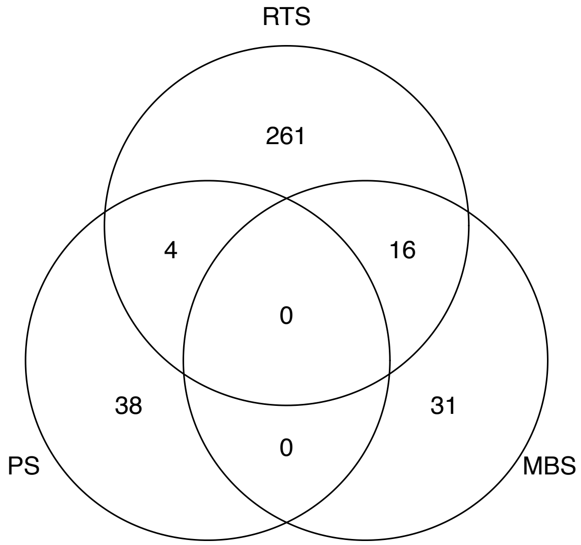

Supplementary Fig. 16: Intersection of the genes found in our data, part of the three gene expression signatures related to aggressive clinical behavior. No gene is common among all three signatures.

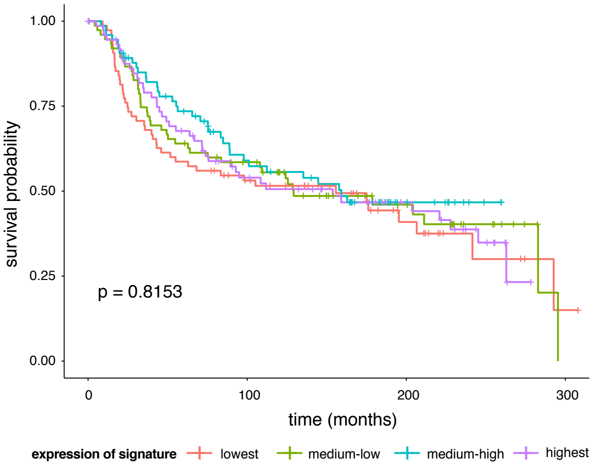

(a) cluster 1

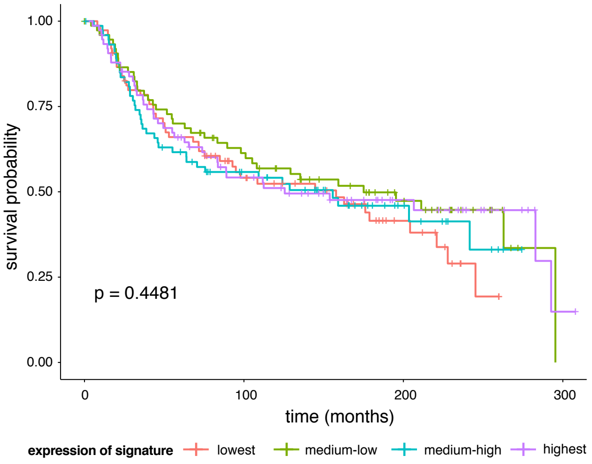

(b) cluster 3

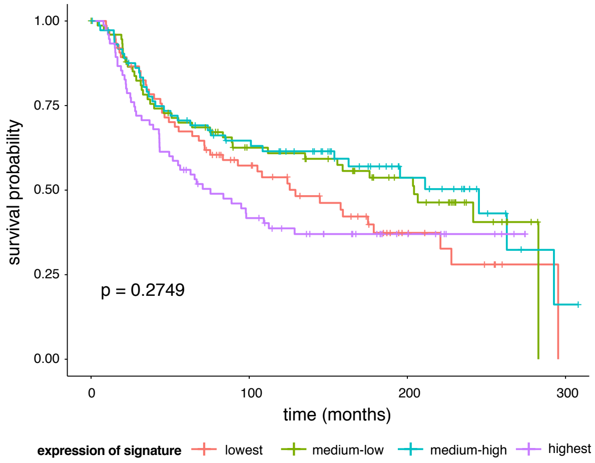

(c) cluster 4

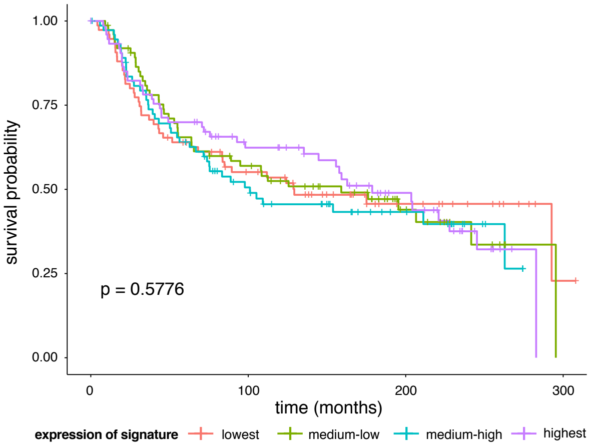

(d) cluster 5

Supplementary Fig. 17: Kaplan Meier survival curves for the top 10 differentially expressed genes in clusters 1,3,4 and 5, as compared to all other epithelial cells, on the METABRIC dataset (Pereira et al., 2016) (p-value from log-rank test). The top 10 differentially expressed genes have been identified similarly to the cluster2-related signature. Separation on quartiles is for visualization purposes.

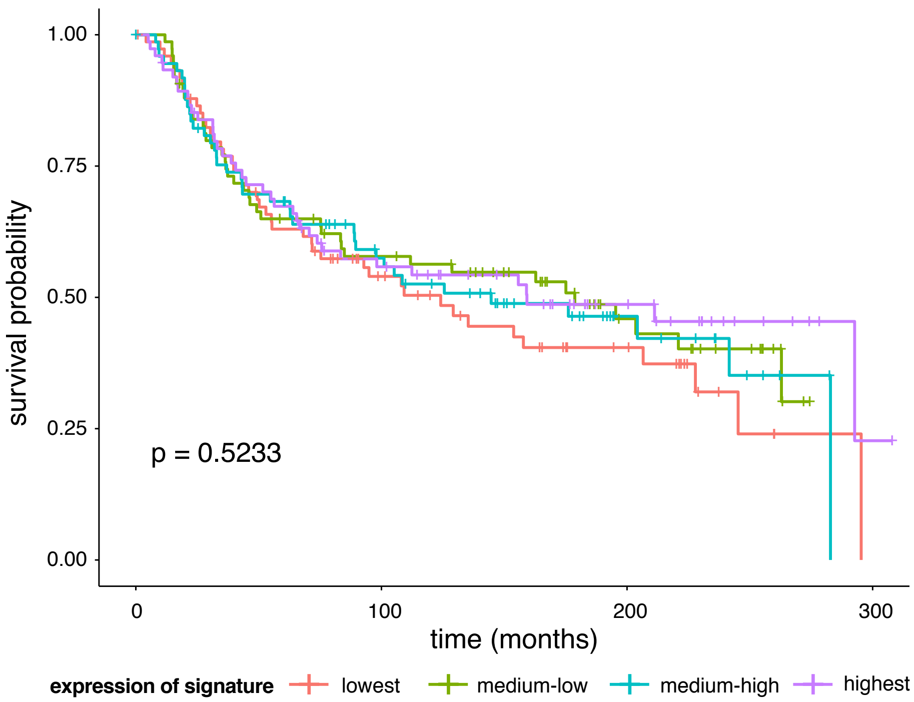

Supplementary Fig. 18: Kaplan Meier survival curves for the intrinsic basal signature (Sørlie et al., 2001), on the METABRIC dataset (Pereira et al., 2016) (p-value from log-rank test). Separation on quartiles is for visualization purposes.

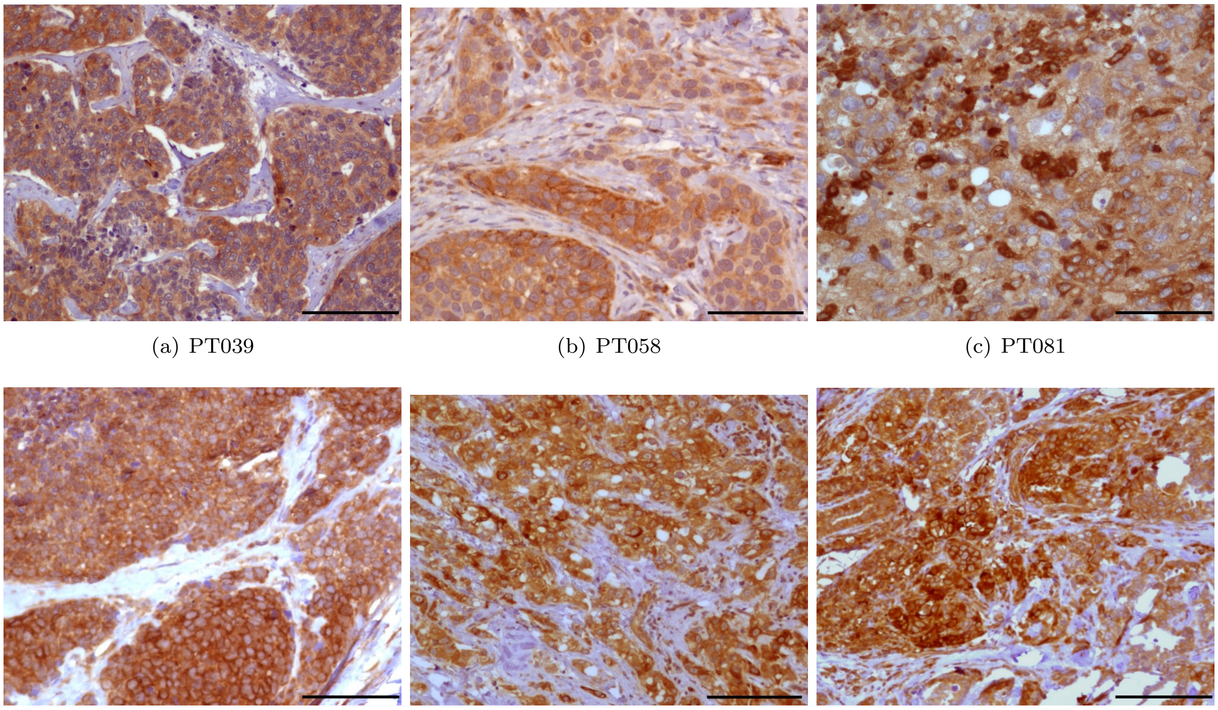

(a) PT039 (b) PT058 (c) PT081

(d) PT084 (e) PT089 (f) PT126

Supplementary Fig. 19: Immunohistochemical analysis for SPTLC1 of the six primary TNBCs analyzed in this study, showing intense staining in a subset of tumor cells in each case. Scale bar represents 50 um.

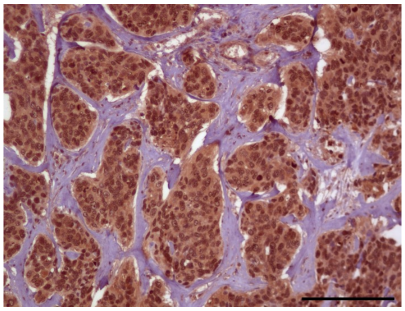

(a) PT039

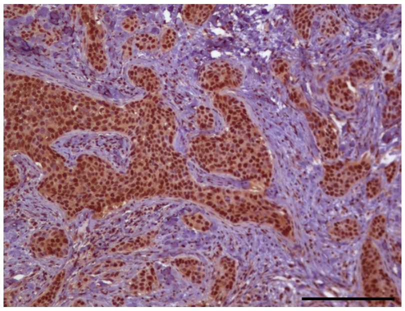

(b) PT058

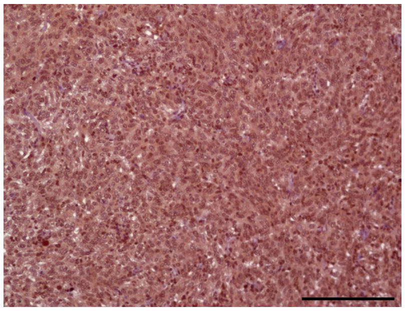

(c) PT081

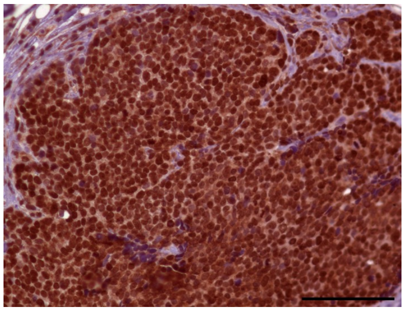

(d) PT084

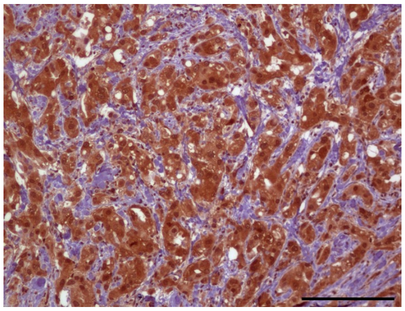

(e) PT089

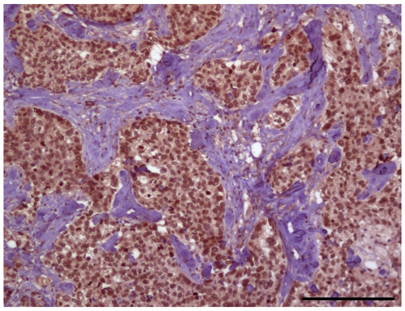

(f) PT126

Supplementary Fig. 20: Immunohistochemical analysis for S1PR1 of the six primary TNBCs analyzed in this study, showing intense expression of sphingosine-1-phosphate receptor 1 indicating activated glycosphingolipid metabolism signaling in tumor cells in each case. Scale bar represents 50 um.

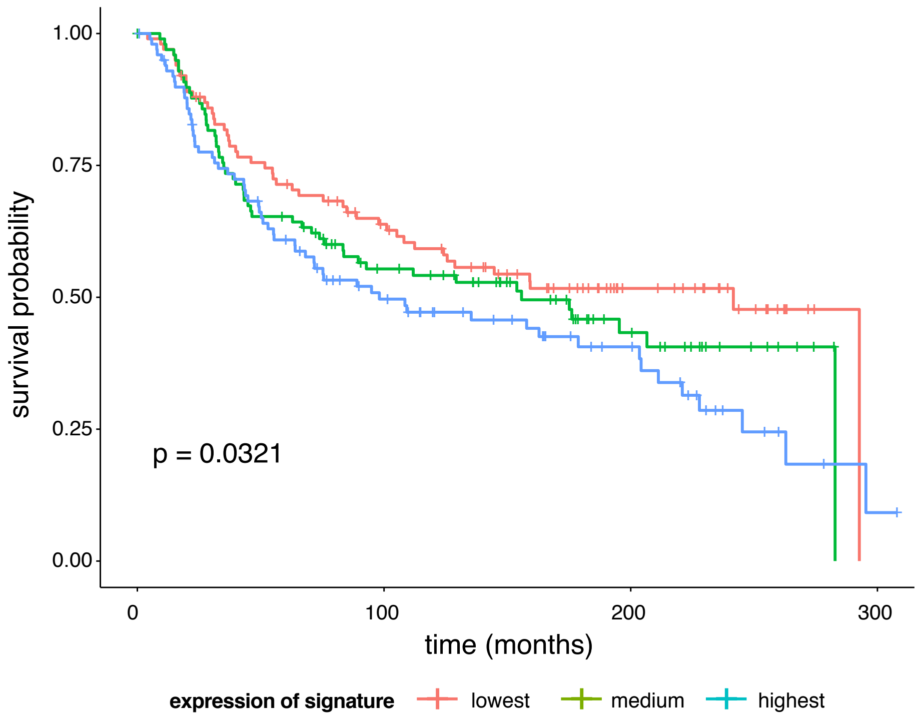

Supplementary Fig. 21: Kaplan Meier survival curves for the glycosphingolipid metabolism pathway (Table 4), on the METABRIC dataset (Pereira et al., 2016) (p-value from log-rank test). Separation on tertiles is for visualization purposes.

Supplementary Tables

Supplementary Table 1: TNBC patient characteristics.

Patient ID	Age	Tumor Size (cm)	Tumor Grade	ER %	PR %	HER2 (IHC Score)	Ki67 (IHC Score)	Lymphovascular Invasion (Y/N)	Nodal Involvement (Y/N)	Genetic Testing
039	64	9.5	3	0	0	0/1+	35 %	N	N	BRCA-
058	46	1.5	3	0	0	2+	$< 10\%$	Y	N	NA
081	45	2.3	3	1-10, 0	< 1, 0	0, 0	$> 50\%$	N	N	BRCA-
084	52	2.3	3	0	0	0/1+	50%	Y	Y (1 node)	BRCA2+
089	44	1.5	2	0	0	0/1+	15%	N	N	BRCA-
126	42	1.7	3	1-10	0	1+	65%	Y	Y (1 node)	BRCA2 VUS

Patient ID	cluster	p-value Wilcoxon
039	2	**1.56e-08**
	3	0.99
	4	0.65
081	2	**0.00017**
	3	0.68
	4	0.53
084	2	**0.00062**
	3	0.74
	4	0.52
089	2	0.89
	3	0.012
	4	0.17

Supplementary Table 2: Associations between the copy number values of genes differentially and highly expressed between cells in each clusters versus all other epithelial cells. One-sided Wilcoxon rank sum test with continuity corrections was conducted to see if the mean segment copy number of these genes is significantly higher than the mean segment copy number of all other genes, for each cluster. The genes characteristic for each cluster were selected as the ones differentially expressed and with highest mean value in the cells pertaining to that respective cluster.

term	p-value
**Lysosome**	0.008
Apoptosis	0.011
p53 signaling pathway	0.016
**Glycosphingolipid biosynthesis - lacto and neolacto series**	0.018
**NOD-like receptor signaling pathway (Innate Immunity)**	0.021
Metabolic pathways	0.029
Pathways in cancer	0.037
**Cytokine-cytokine receptor interaction**	0.038
Biosynthesis of antibiotics	0.041
Signaling pathways regulating pluripotency of stem cells	0.049

Supplementary Table 3: Functional pathways associated with the top 1,000 differentially expressed genes in cluster 2 as compared to all other epithelial cells, for a corrected p-value cutoff of 0.05. The analysis was conducted using the tool David (Huang et al., 2009) and the KEGG database (Kanehisa and Goto, 2000).

gene
ST3GAL4
ST3GAL6
ST8SIA1
FUT1
FUT2
FUT3
FUT4
FUT5
FUT6
FUT7
FUT8
FUT9
SPTLC1
ASAH2
GBA
SMPD1
ARSB
ARSG

ARSI
PRKD3
KDSR
SPHK2
GLA
SMPD2
STS
ESYT1
ARSH
PRKD2
SPTLC2
CERK
GLB1
UGCG
ARSD
SUMF2
ALDH3B1
ORMDL2
CERS1
CERS4
ENPP7
UGT8
ARSE
GLTP
ALDH3B2
FA2H
DEGS2
PLPP1
ACER2
SGPP1
ARSF
ESYT2
ALDH3A2
ORMDL1
SGPP2
SPHK1
ARSA
DEGS1
B4GALNT1
GBA3
CSNK1G2
ORMDL3
SGMS2
CERS5
NEU1
PLPP2
GM2A
GLB1L
GDF1
PPM1L
CERS3
NEU3
NEU2
PLPP3
HEXA
ARSJ

OSBP
SPTSSB
CERS6
ACER1
ACER3
SGPL1
HEXB
CPTP
PRKD1
SERINC1
CERS2
NEU4
SMPD3
SPTSSA
CTSA
ESYT3
VAPB
ASAH1
GALC
SMPD4
B4GALT6
PSAP
ARSK
VAPA
SPTLC3
SGMS1
GBA2
GAL3ST1
B3GALNT1
SUMF1
COL4A3BP

Supplementary Table 4: The genes part of the glycosphingolipid metabolism pathway.

Supplementary Methods

Biological samples processing

Human tumor specimens

Fresh tumors from TNBC specimens (Table 1) were collected at Massachusetts General Hospital with approval by the Institutional Review Board (93-085). Five of six patients underwent genetic testing, revealing that patient PT084 was a BRCA2 mutation carrier and the others lacked BRCA1/2 mutation, while patient PT058 did not undergo such testing. Tumor tissues were mechanically and enzymatically dissociated using a tumor dissociation kit (Miltenyi Biotec). Single cell suspensions were collected after removing large pieces of debris with a 40 μm cell strainer.

Flow cytometry and sorting

Tumor cells were blocked in 3% FBS in Hanks buffered saline solution, and then stained first with CD45-Vioblue direct conjugate antibody (Miltenyi Biotec). Cells were washed and then stained for viability (Calcein AM, TO-PRO-3, Life Technologies). FACS was performed on FACSAria Fusion (Becton Dickinson). Strict singlets were selected by using standard criteria for forward scatter height versus area. Viable cells were identified by staining positive with Calcein (FITC) and negative for TO-PRO-3 (APC). Single cells were sorted into 96 well plates containing 10 μl TCL buffer (Qiagen) + 1% β-mercaptoethanol. Plates were spun briefly, snap-frozen on dry ice immediately, and then stored at $-80°$ C till further processing.

cDNA synthesis, library construction and sequencing

Smart-seq2 was performed on single sorted cells (Picelli et al., 2014), with the following modifications: RNA was cleaned up using Agencourt RNAClean XP beads (Beckman Coulter). Reverse transcription was carried out using oligo-dT primers, Maxima reverse transcriptase and locked TSO oligonucleotide prior to PCR amplification with KAPA HiFi HotStart ReadyMix (Kapa Biosystems). Subsequently, Agencourt AMPure XP bead (Beckman Coulter) purification was applied. Full length cDNA libraries were barcoded using the Nextera XT Tagmentation protocol (Illumina). Libraries from 96 pooled cells were sequenced as 38 bp paired end on NextSeq 500 (Illumina).

mRNA data preprocessing

Expression quantification and alignment

The single-cell RNASeq (scRNA-seq) samples were sequenced as paired-end reads, with constant read length of 38 base pairs (bps). FASTQ files were quantified to transcript per million (TPM) expression values with RSEM (Li and Dewey, 2011) with default parameters, using the reference genome version GRCh38. We chose to primarily use RSEM expression values for our subsequent analyses, as opposed to *e.g.* STAR counts (Dobin et al., 2013), due to their superior accuracy, as demonstrated by comparative simulation studies (Bray et al., 2016; Teng et al., 2016).

Quality control

In general, scRNA-seq data is expected to be noisier than bulk RNA-Seq data, mainly because of the challenge to reliably convert and amplify the low amount of RNA in a single cell (also known as *RNA capture*). Thus, *dropout* events, *i.e.* the situation in which either no expression or low expression levels are recorded in a gene across many cells, not necessarily reflective of the true biological signal, are frequent in scRNA-seq data. Not removing either low quality cells or genes with too low expression can bias downstream analyses, making it more difficult to separate biological signal from technical or biological noise.

Removal of low quality cells

The dataset initially consisted of 1,529 single cells pertaining to 6 TNBC patients, and of 7 pooled samples of 100 cells each. We use the following metrics to identify low quality cells (Supplementary Fig. 22):

1. **Library size**: library size is the number of mapped reads per cell. As a cell is sequenced at a deeper level, the expression quantification is more precise. Cells with relatively small library sizes generally indicate that the RNA has not been efficiently captured during library preparation.

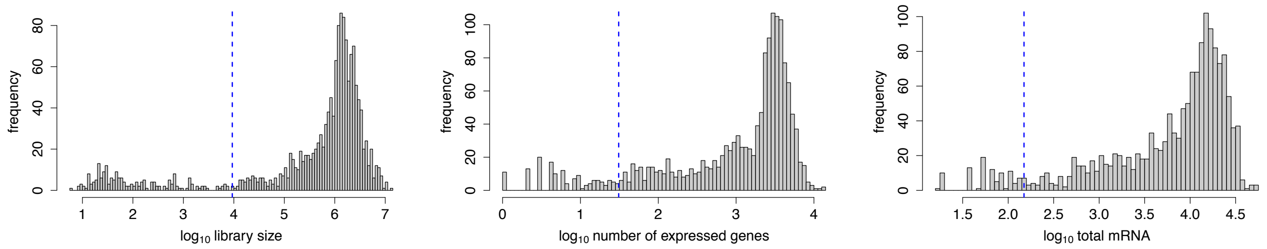

(a) Library size per cell.

(b) Number of expressed genes per cell. A gene is considered expressed if its $\log_2(\text{TPM}+1) > 0.1$.

(c) Total amount of mRNA per cell, quantified as the sum of the $\log_2(\text{TPM}+1)$ values for all genes in each cell.

Supplementary Fig. 22: Quality control of single cells. Blue dashed vertical lines represent the data-derived thresholds, 4 MADs below the median. The log-transformation improves resolution at small values, particularly if the MADs of the raw values are greater than the median (Lun et al., 2016b). After filtering, $1,326$ cells pass all three thresholds.

2. **Number of expressed genes**: cells with very few expressed genes are poor indicators of the true cellular transcriptional diversity. A gene is considered expressed if its $\log_2(\text{TPM}+1) > 0.1$.

3. **Total amount of mRNA**: the total amount of mRNA is quantified as the sum of the $\log_2(\text{TPM}+1)$ values for all genes in each cell. Cells with too low amount of mRNA indicate either unsuccessful capture, or dead or compromised cells.

We assume that our dataset consists mainly of high quality cells and we remove all cells that are at least 4 median absolute deviations (MADs) below the median for any of the above metrics, as discussed in (Lun et al., 2016b). The rationale behind this filtering procedure is choosing adaptive and unbiased data-derived thresholds. $1,326$ cells pass all three thresholds, among which 5 are pooled samples.

Removal of unexpressed genes

For each of the 6 patients, we identify the genes that are not expressed ($\log_2(\text{TPM}+1) < 0.1$) in at least 95% of cells for that respective patient, and remove the intersection of these 6 sets. Requiring for the genes labeled as unexpressed to be unexpressed in each patient, rather than only across all patients, increases our chance of retaining genes related to inter-patient heterogeneity. From the initial $21,785$ genes, $13,280$ remain after filtering.

Normalization

Normalization is an essential step in preprocessing scRNA-seq data, especially given the large influence that various confounding factors have been found to have on the quantified expression levels in single cells (McCarthy et al., 2016; Hicks et al., 2015). Examples of confounding factors include the frequency of dropout events, the low amount of mRNA in a single cell, high variability between the capture of different cell types, or technical noise (Brennecke et al., 2013; Vallejos et al., 2017). Our normalization strategy consists of the following three steps:

1. Transform the TPM values into *relative counts* with the Census algorithm (Qiu et al., 2017) (function `relative2abs` from the R package `monocle` (Qiu et al., 2017)). Census rescales the TPM values in each cell by dividing them by the estimated total number of mRNA molecules per cell. In this way, the TPM values are transformed into relative Census counts, which are negative-binomially distributed. These counts have been shown (Qiu et al., 2017) to better reflect the actual amount of mRNA in single cells, as compared to either TPMs alone or counts normalized with other approaches, such as edgeR (Robinson et al., 2010), DESeq2 (Love et al., 2014) or SCDE (Kharchenko et al., 2014).

2. Normalize the Census counts with the deconvolution strategy implemented in the R package `scran` (Lun et al., 2016a). `scran` computes cell-specific scaling factors (also known as *size factors*, *i.e.* random variables modeling nuisance effects), while specifically considering the high dropout rate of scRNA-seq. Dividing the expression of each cell by its size factor is meant to diminish the nuisance effects (Vallejos et al., 2017). The

resulting values are further log2-transformed, after adding a constant offset of 1. 137 of the cell-specific size factors are estimated to be 0, and are considered to correspond to cells with too low transcriptomic diversity. These cells are removed from the dataset, and 1, 189 single cells remain for downstream analyses (Table 5).

3. Remove additional sources of unwanted variation in the `scran`-normalized Census counts with `RUVSeq` (Risso et al., 2014) (`RUVg`). `RUVg` uses housekeeping genes considered to have approximately constant expression across samples, and regresses out the variation estimated from the expression of the housekeeping genes, using a generalized linear model. `RUVg` requires as input the number of sources of variation to be removed (k). Here, as the data has been normalized in two previous steps, we set $k = 1$. We use the curated list of 98 housekeeping genes compiled in (Tirosh et al., 2016), of which 93 genes are present in our dataset (Supplementary Fig. 23). Note that this normalization step can result in negative expression values.

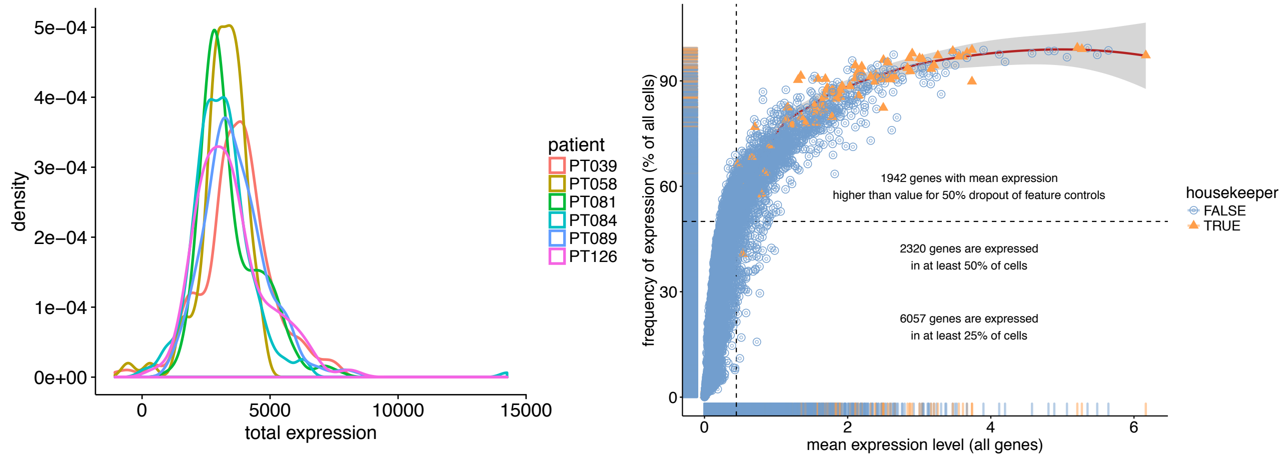

(a) Distribution of total expression per patient, after normalizing the data as described above.

(b) Mean and frequency of expression for all 13, 280 genes, colored by whether they are housekeepers or not, with a smoothed fit and 1 standard deviation shown.

Supplementary Fig. 23: Gene expression statistics in the normalized dataset.

patient	PT039	PT058	PT081	PT084	PT089	PT126
cells	281	78	225	230	286	89

Supplementary Table 5: Number of single cells per patient, after quality control and normalization (total: 1, 189 cells).

From here onwards, we will refer to the data on which the three-step normalization procedure described above has been applied, as *normalized data*. We compare the effectiveness of different normalization strategies using the cell-wise relative log expression metric (RLE). For each cell, RLE is the median across genes of the log ratio of its expression, to the median expression of that gene across cells. An offset of 1 is added to both the numerator and denominator before taking the log. Normalized cells are expected to have RLEs close to 0, while unnormalized cells are expected to significantly deviate from 0 (cells with many reads are expected to have positives RLEs, and cells with few reads negative RLEs). We compare RLE levels (Supplementary Fig. 24) between the normalized data (*normalized*), the raw TPM data (*TPM*), and data normalized with either only Census (*Census counts*, corresponding to normalization step 1 above), `scran` (*scran counts*, corresponding to normalization step 2 above applied on the raw read counts), or `scran` applied on the Census counts (*scran Census counts*, corresponding to normalization steps 1 and 2 above). The *scran Census counts* scenario is the most similar to the normalized data, missing however the removal of unwanted variation with `RUVg` (Risso et al., 2014). *normalized* data and *scran Census counts* have the lowest variation in RLEs (with *scran Census counts* showing slightly less variability in the majority of cells, but more extreme outliers, than the *normalized* data), indicating that technical variability has generally been removed from the data.

When evaluating various normalization methods (Supplementary Figs. 25-28), we didn't include methods specific to bulk RNASeq data, such as edgeR (Robinson et al., 2010) or DESeq2 (Love et al., 2014), since they have been

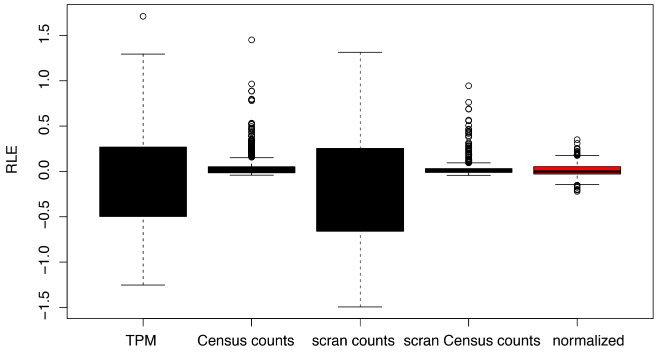

Supplementary Fig. 24: RLEs (cell-wise relative log expression) across all cells, for various normalization strategies. All expression levels are $\log_2$-transformed, after adding an offset of 1. The normalization strategy we are using for downstream analyses in this manuscript is highlighted in red.

shown to perform more poorly on scRNA-seq data as compared to single-cell specific methods (Vallejos et al., 2017).

The number of genes expressed and the library size per cell are well-known confounding factors in scRNA-seq data (Hicks et al., 2015; Tirosh et al., 2016), and we identify these relationships in our dataset prior to normalization as well. After normalizing, the effects of these confounders are significantly reduced, while alternative normalization methods achieve very modest such reductions (Supplementary Fig. 25). More specifically, even though the number of genes expressed correlates with the first principal component for $log_2(TPM{+}1)$ ($R^2 = 0.68$), $log_2(Census\ counts{+}1)$ ($R^2 = 0.81$), $log_2(scran\ counts{+}1)$ ($R^2 = 0.67$), and $log_2(scran\ Census\ counts{+}1)$ ($R^2 = 0.37$), it doesn't correlate with the first component for the normalized data ($R^2 < 0.001$, Supplementary Fig. 26). Rather, following the three-step normalization, the number of genes expressed correlates most with the third principal component and only accounts for $R^2 = 0.18$ of the variance in expression explained by this component. In the case of the raw counts, the second principal component correlates most with the number of genes expressed, while the first component correlates most with the library size ($R^2 = 0.66$, Supplementary Fig. 25).

After normalization, the patient correlates with the first PCA component ($R^2 = 0.45$, Supplementary Fig. 27), while in all alternative normalization approaches, including raw counts, the patient correlates at most with the second PCA component. `RUVg` removes the noise from the data explained by the first principal component, leaving the highest amount of variation to be explained by the patient. However, the effect of the sequencing batch remains strong after normalization, even after removing sources of unwanted variation (Supplementary Fig. 28). Normalizing specifically for the batch effect would nevertheless imply removing also part of the patient-related heterogeneity, as two patients are perfectly confounded with two sequencing batch (Table 6).

In the normalized data, cells cluster relatively well by patient in the non-linear framework of t-SNE (Maaten and Hinton, 2008), and less well in the linear PCA framework (Supplementary Fig. 29). The dropout rate (the percentage of unexpressed genes per cell) is at least 50% in the large majority of cells (Supplementary Fig. 30), as previously reported in scRNA-seq studies (Hicks et al., 2015). All subsequent analyses were performed on the normalized data.

Supplementary Table 6: The patients corresponding to each sequencing batch.

batch	patient
B1	PT039
B2	PT058
B3	PT039, PT081, PT089
B4	PT081
B5	PT084, PT089
B6	PT084, PT089
B7	PT126
B8	PT039, PT084
B9	PT039

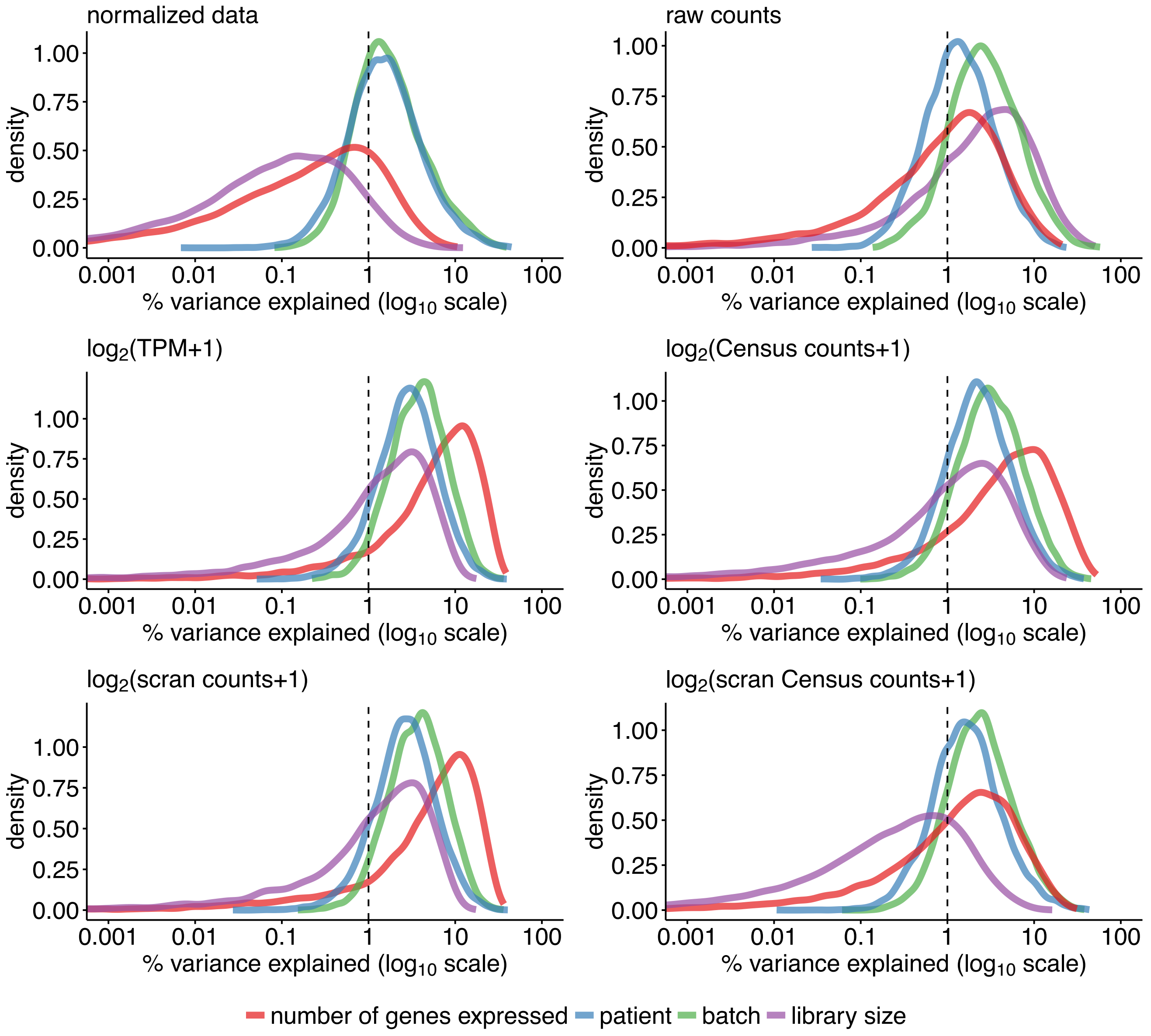

Supplementary Fig. 25: Percentage of phenotypic variance explained by various variables, as measured by R^2 in a marginal linear model regressing expression values for each gene separately against each variable. For each variable, the density of the gene-wise marginal R^2 values is plotted, as implemented in `scater` (McCarthy et al., 2016). We compare the above-described normalization strategy (*normalized data*), *raw counts*, and four additional normalization strategies: $log_2(TPM{+}1)$ are the counts only normalized by library size, $log_2(Census\ counts{+}1)$ are the Census counts and correspond to only step 1 of our normalization procedure, $log_2(scran\ counts{+}1)$ are the `scran`-normalized raw counts, corresponding to normalization step 2 above applied on the raw counts, and $log_2(scran\ Census\ counts{+}1)$ are the scran-normalized Census counts, corresponding to normalization steps 1 and 2 above. The library size explains the highest percentage of phenotypic variance for raw counts, and the number of genes expressed is the strongest explainer for variance in TPM values. Its effect is strongly reduced after the three-step normalization, but remains high after normalizing with either only Census or `scran`, or with the combination of the two. The effect of the sequencing batch (Table 6) is higher than the patient effect in almost all instances, except for the normalized data, when the two become almost indistinguishable.

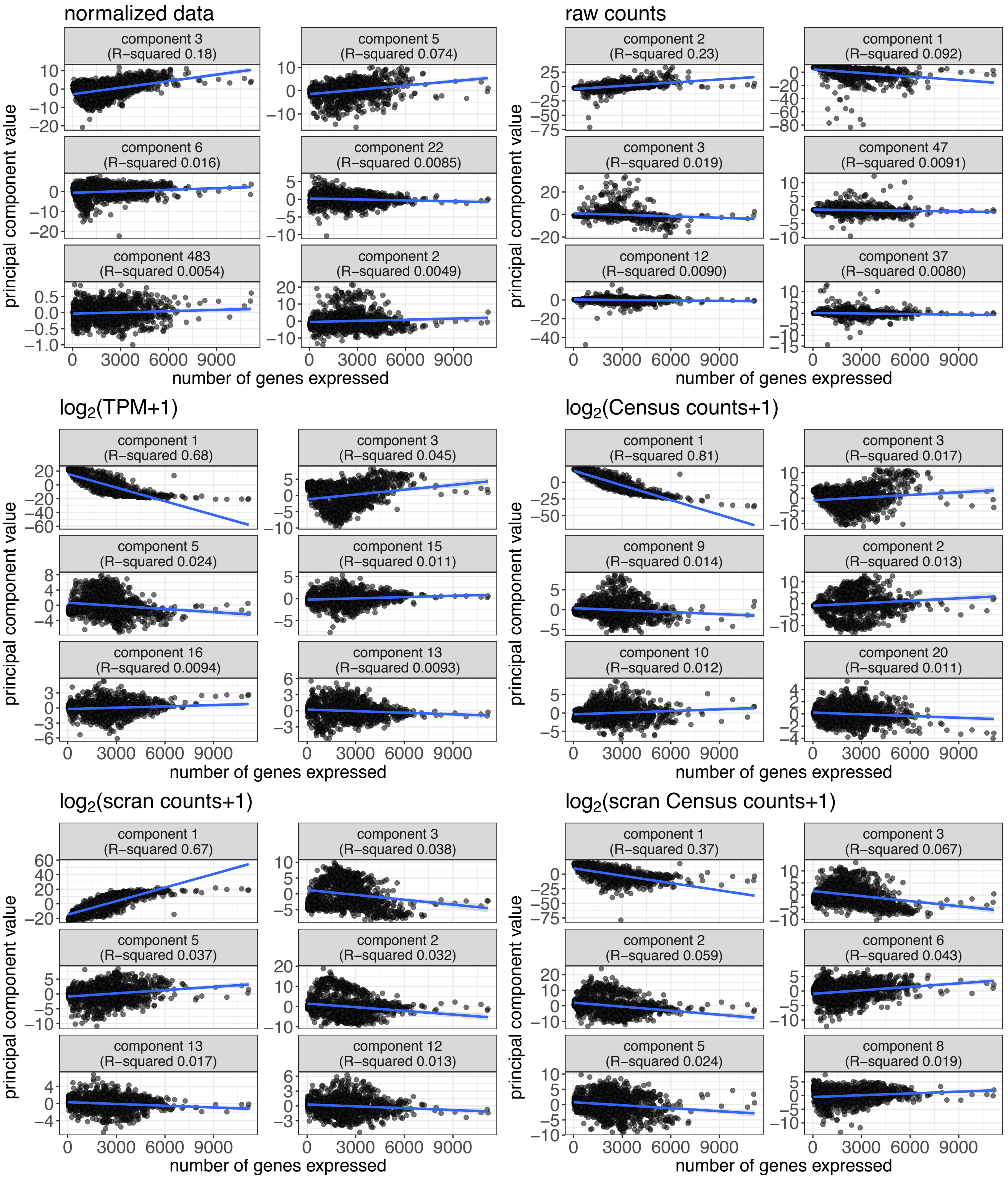

Supplementary Fig. 26: The six most important principal components that correlate with the number of genes expressed, ranked by R^2 values from a linear model regressing each principal component onto the number of genes expressed. In computing the principal components, the 500 most variable genes have been used, as implemented in `scater` (McCarthy et al., 2016). After normalization, the number of genes expressed does not correlate with the first principal component anymore, and it only weakly correlates with the third PCA component ($R^2 = 0.18$). Before normalization (TPM data) or under alternative normalization strategies (Census counts, `scran` counts, and `scran` Census counts), the number of genes expressed strongly correlates with the first PCA component (R^2 between 0.37 and 0.81). As expected and as seen in Supplementary Fig. 25, the highest percentage of variation in raw counts in explained by library size, which correlates most with the first principal component ($R^2 = 0.66$, plot not shown).

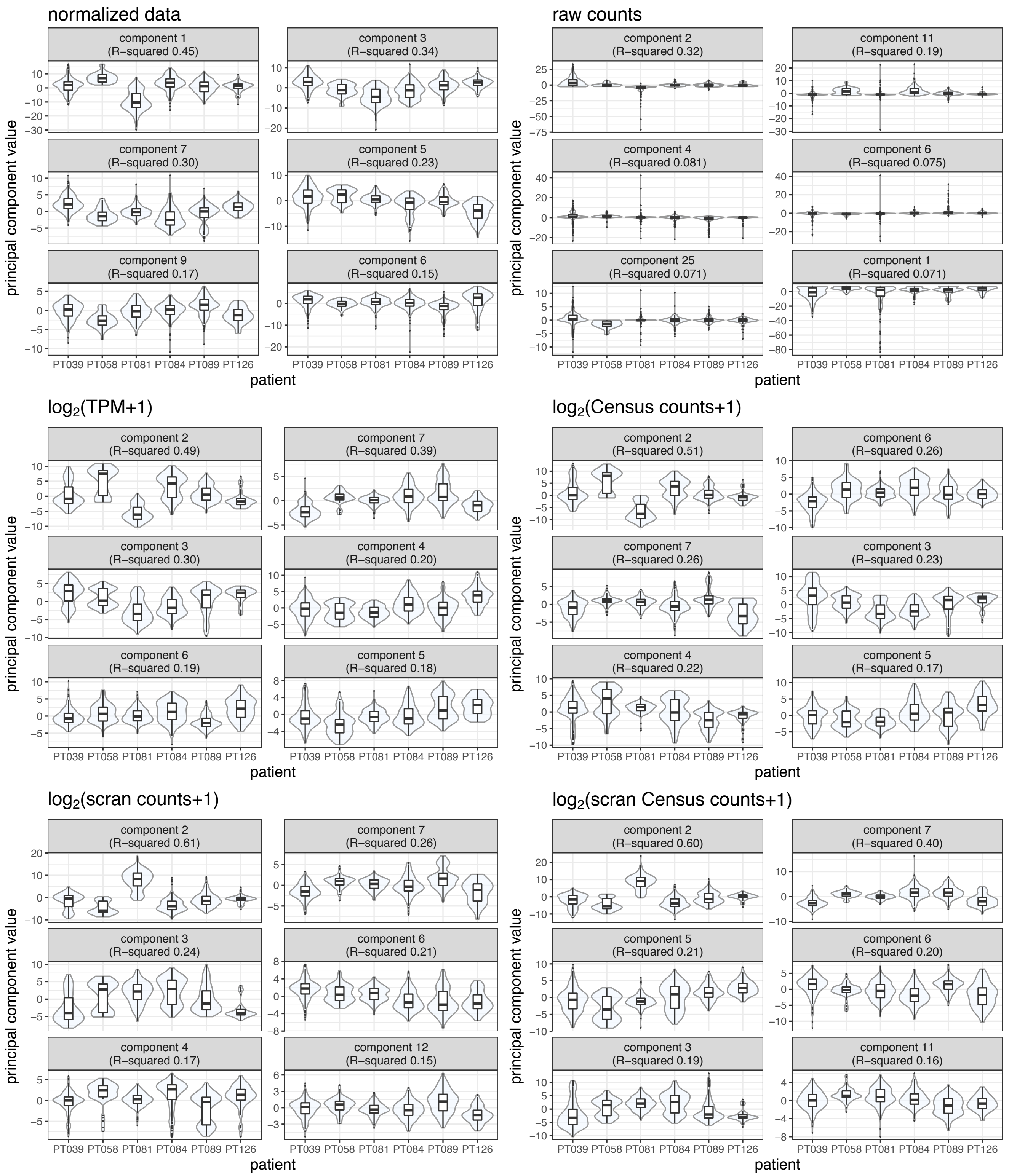

Supplementary Fig. 27: The six most important principal components that correlate with the patient, ranked by R^2 values from a linear model regressing each principal component onto patients. In computing the principal components, the 500 most variable genes have been used, as implemented in `scater` (McCarthy et al., 2016). In the normalized data, the patient correlates with the first PCA component ($R^2 = 0.45$), while in all other cases the patient correlates with only the second PCA component.

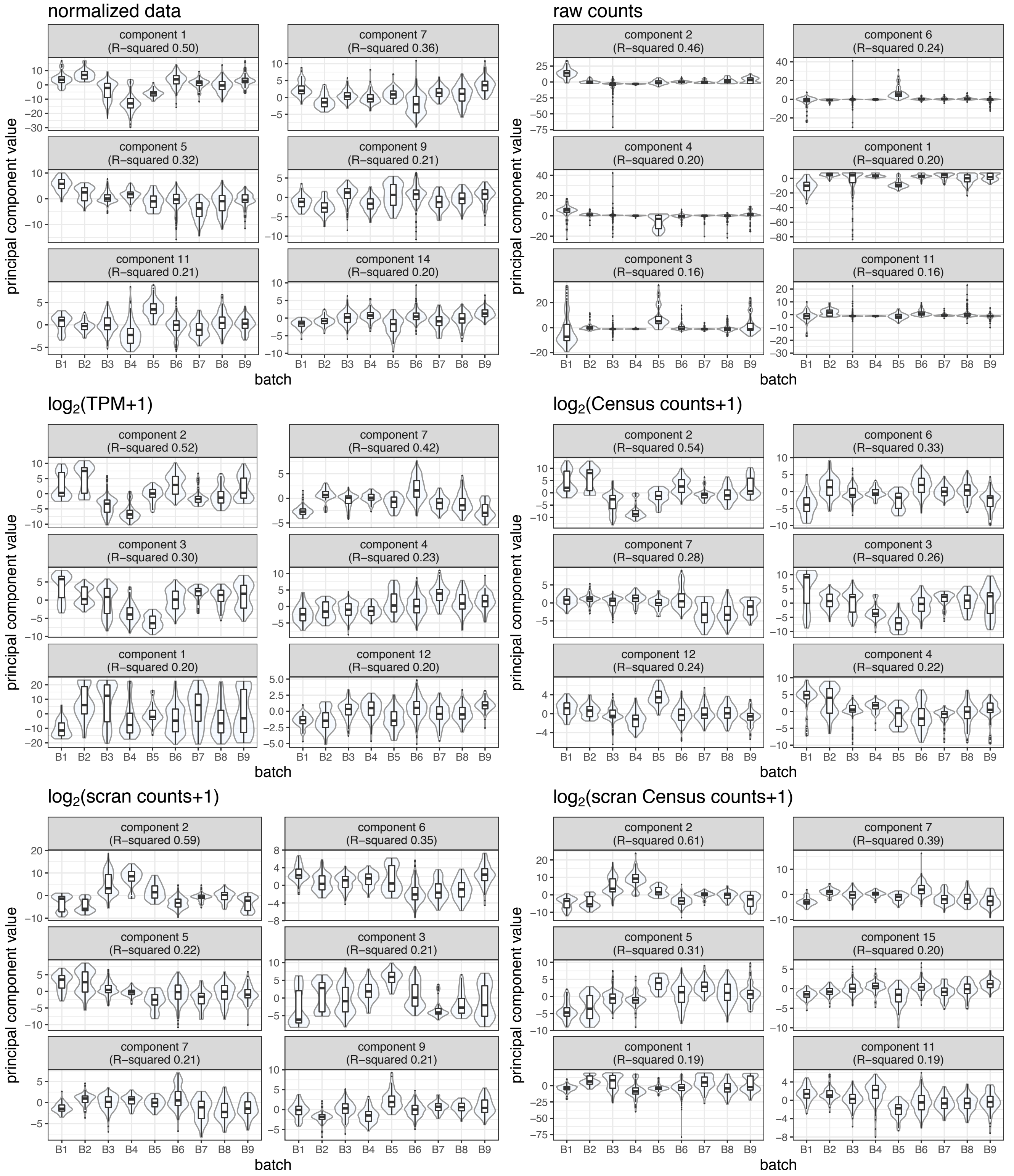

Supplementary Fig. 28: The six most important principal components that correlate with the sequencing batch, ranked by R^2 values from a linear model regressing each principal component onto sequencing batches. In computing the principal components, the 500 most variable genes have been used, as implemented in `scater` (McCarthy et al., 2016). Similar to the patient effect, the sequencing batch correlates with the first PCA component in the normalized data ($R^2 = 0.50$), while in all other cases it correlates with only the second PCA component.

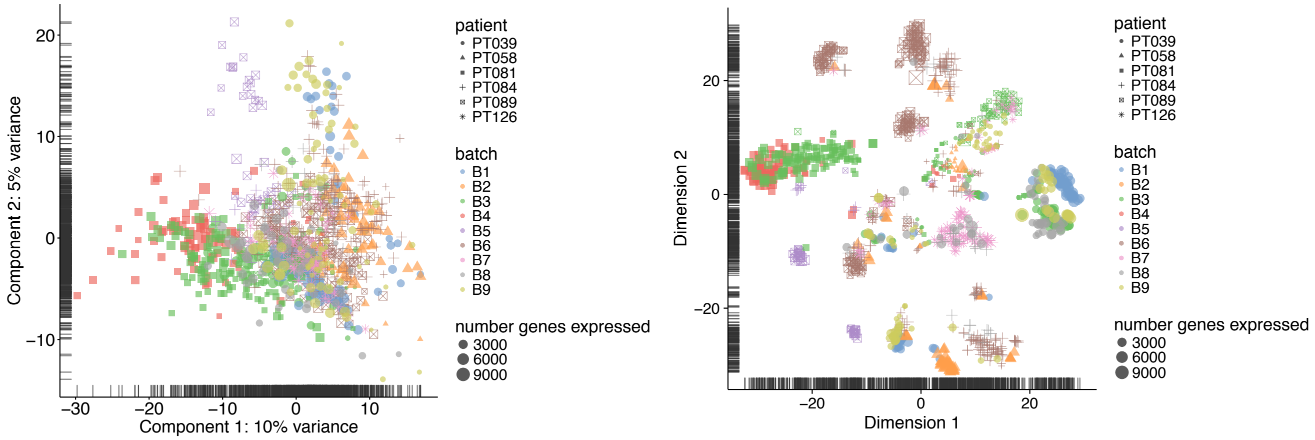

Supplementary Fig. 29: PCA and t-SNE on the normalized data. For PCA, the top 500 most variable genes were used. For t-SNE, the perplexity was chosen to be 30.

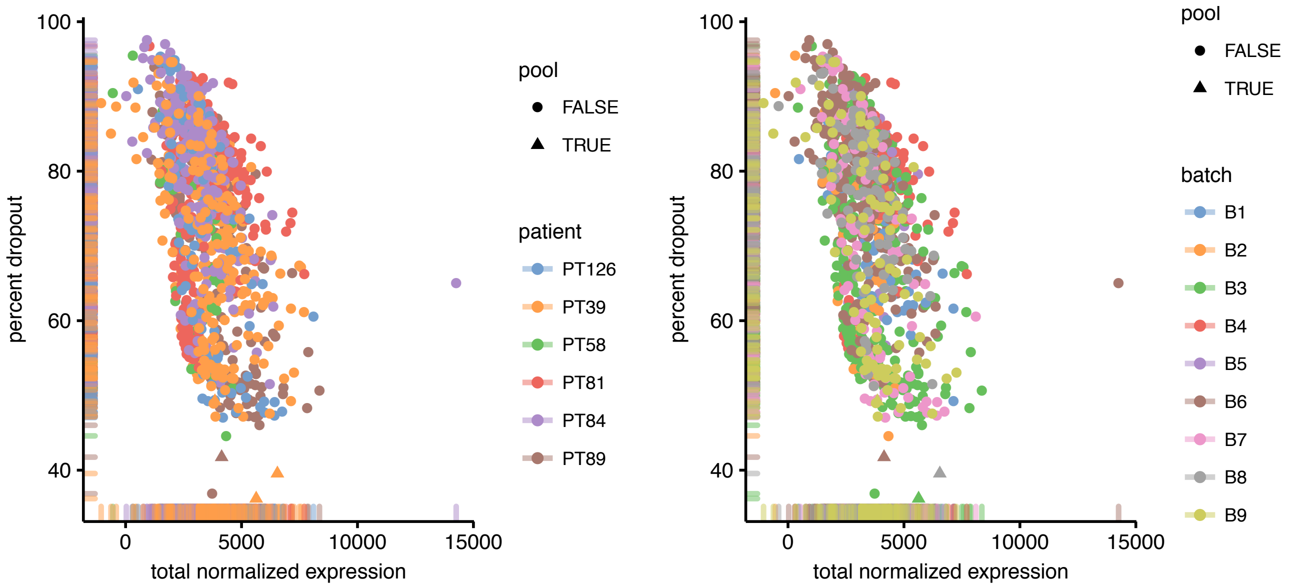

Supplementary Fig. 30: The total normalized expression and the percentage of dropout events (defined as the percentage of unexpressed genes per cell) in each of the cells, colored by either patient or sequencing batch. In the large majority of cells, at least 50% of genes are unexpressed. Triangles represent pools of 100 cells.

Identifying cell types

Given the high dropout rate of scRNA-seq data and the high variability in cell type identification of the various algorithms developed for this task (Freytag et al., 2017), we employed a two-step combination approach to identify the different cell types that tumors consist of:

1. Literature-based list of specific expression markers previously established to define cell types;
2. Clustering.

Expression markers

The literature-based list used to classify cell types consists of 49 expression markers specific to four cell types, compiled from multiple references (Tirosh et al., 2016) (Table 7). In order to minimize the number of misassignments, we only assign cells to a specific cell type when we consider that there is enough expression-based evidence supporting the assignment (expression threshold = 1). To this end, we derive a series of expert-based rules:

Supplementary Table 7: Markers used for classifying the single cells into different cell types.

Marker	type	subtype
EPCAM	epithelial	
EGFR	epithelial	
CDH1	epithelial	
KRT14	epithelial	basal epithelial
ITGA6	epithelial	basal epithelial
KRT5	epithelial	basal epithelial
TP63	epithelial	basal epithelial
KRT17	epithelial	basal epithelial
MME	epithelial	basal epithelial
KRT8	epithelial	luminal epithelial
KRT18	epithelial	luminal epithelial
KRT19	epithelial	luminal epithelial
FOXA1	epithelial	luminal epithelial
GATA3	epithelial	luminal epithelial
MUC1	epithelial	luminal epithelial
CD24	epithelial	luminal epithelial
KIT	epithelial	luminal progenitor
GABRP	epithelial	luminal progenitor
FAP	stroma	
COL1A1	stroma	
COL3A1	stroma	
COL5A1	stroma	
ACTA2	stroma	
TAGLN	stroma	
LUM	stroma	
FBLN1	stroma	
COL6A3	stroma	
COL1A2	stroma	
COL6A1	stroma	
COL6A2	stroma	
PECAM1	endothelial	
VWF	endothelial	
CDH5	endothelial	
SELE	endothelial	
PTPRC	immune	
CD2	immune	T cell
CD3D	immune	T cell

CD3E	immune	T cell
CD3G	immune	T cell
CD8A	immune	T cell
CD8B	immune	T cell
MS4A1	immune	B cell
CD79A	immune	B cell
CD79B	immune	B cell
BLNK	immune	B cell
CD14	immune	macrophage
CD68	immune	macrophage
CD163	immune	macrophage
CSF1R	immune	macrophage

1. **Epithelial class**: a cell has epithelial characteristics if it expresses either:
   - at least 2 epithelial markers;
   - only one of the strongest breast epithelial markers: *EPCAM*, *KRT8*, *KRT18*, *KRT19*, with expression higher than in 50% of the cells for that respective patient, for that marker.
2. **Specific immune class**: a cell has specific immune characteristics (T cell, B cell, macrophage) if it expresses either:
   - only specific immune markers of that type (at least 2);
   - *PTPRC* and only specific immune markers of that type (at least 1);
   - at least 3 immune markers of that type, and at most 1 immune marker of another type.
3. **Stroma class**: a cell has stroma characteristics if it expresses either:
   - only stroma markers;
   - at least 3 stroma markers and at most 1 endothelial marker.
4. **Endothelial class**: a cell has endothelial characteristics if it expresses either:
   - only endothelial markers;
   - at least 3 endothelial markers and at most 1 stroma marker.

After this first step, the cells that are not assigned to any of the above categories are labeled as *unknown*, and the cells that belong to more than one of the above categories are labeled as *undecided*. Exceptionally, the cells that express both epithelial and stroma markers are considered to indicate the epithelial-mesenchymal transition (*EMT*) state and are assigned to the epithelial class. In addition to the markers in Table 7, we also test whether any of the single cells are natural killers or dendritic cells, using the markers suggested in (Tirosh et al., 2016), without however identifying any such cells in our dataset. Following this classification step, the resulting distribution of cells per cell type is shown in Table 8.

cell type	epithelial	stroma	macrophage	T cell	B cell	endothelial	undecided	unknown
number of cells	831	95	51	53	19	14	31	95

Supplementary Table 8: Number of single cells per cell type, after classifying cells based solely on specific expression markers (total: 1,189 cells).

Clustering

Nevertheless, classifying cells based on expression markers alone can be imprecise, particularly given the high dropout rate of scRNA-seq data. Therefore, we next apply t-SNE-based clustering on a projection of the cells into a lower dimensional space, as implemented in `Monocle` (Qiu et al., 2017) and inspired by `Seurat` (Butler and Satija, 2017). Prior to clustering, we regress out the patient effect, in order for the resulting clusters not to be mainly

represent different patients. In addition, we select a subset of expressed genes (mean expression > 0.1) with high dispersion across cells, in order to increase the robustness of the inferred clusters, as discussed in (Qiu et al., 2017). A dispersion parameter is chosen for each gene by first computing an empirical dispersion value, and then fitting a dispersion-mean relationship, as implemented in `estimateDispersions` in `DESeq` (Love et al., 2014). The chosen genes have the empirical dispersion higher or equal that the dispersion fit. The number of clusters is automatically chosen by `Monocle`, implementing the density-based approach introduced in (Rodriguez and Laio, 2014).

We identify nine clusters in our dataset of 1,189 cells. We refine each cluster by attempting to both assign unknown and undecided cells, and correct previous marker-based assignments, given high similarity of cells in each cluster. Specifically, for each cluster for which at least 80% of the assigned cells are of a single type, we compute the average expression of the unknown and undecided cells part of that cluster for each of the marker classes. If the unassigned cells show highest average marker expression for the prevalent cell type of the cluster, then we assign them. In addition, since these clusters consist of a prevalent cell type, we evaluate whether the average marker expression of the other cell types in the same cluster is highest for the prevalent cell type. In this case, we reassign these cells to the prevalent cell type. Alternatively, for the clusters which don't consist of a single predominant cell type (< 80% of assigned cells), we do not either assign any unknown or unidentified cells, or reassign any cells.

The nine clusters consist of the following cell types:

- **cluster 1**: 52 epithelial cells.
- **cluster 2**: 3 epithelial, 13 macrophage, 1 undecided and 1 unknown cells. We assign as macrophages both unassigned cells, since they have stronger average expression for the immune markers than for any other class of markers. Similarly, we further reassign the 3 epithelial cells as macrophages, since they have strongest average expression for the immune markers. After assignment and reassignment, cluster 2 consists of 18 macrophages.
- **cluster 3**: 46 epithelial, 86 stroma, 14 endothelial, 5 undecided and 5 unknown cells.
- **cluster 4**: 185 epithelial, 3 stroma, 4 undecided and 5 unknown cells. We assign as epithelial 5 of the 9 unassigned cells, since they have stronger average expression for the epithelial markers than for any other class of markers. None of the stroma cells have strong epithelial expression. After assignment, cluster 4 consists of 190 epithelial, 3 stroma, 2 undecided and 2 unknown cells.
- **cluster 5**: 46 epithelial, 4 stroma, 52 T, 3 B, 11 undecided and 28 unknown cells.
- **cluster 6**: 93 epithelial, 1 B and 25 unknown cells. We assign as epithelial 19 of the 25 unassigned cells, since they have stronger average expression for the epithelial markers than for any other class of markers. The B cell doesn't have strong epithelial expression. After assignment, cluster 6 consists of 112 epithelial, 1 B and 6 unknown cells.
- **cluster 7**: 128 epithelial and 1 unknown cells. The unknown cell doesn't have strong epithelial expression.
- **cluster 8**: 277 epithelial, 2 stroma, 1 T, 15 B, 5 undecided and 28 unknown cells. We assign as epithelial 16 of the 33 unassigned cells, since they have stronger average expression for the epithelial markers than for any other class of markers. Among the B, T and stroma cells, only one cell has highest epithelial expression. After assignment and reassignment, cluster 8 consists of 294 epithelial, 1 stroma, 1 T, 15 B, 1 undecided and 16 unknown cells.
- **cluster 9**: 1 epithelial, 38 macrophage, 5 undecided and 2 unknown cells. We assign as macrophages all unassigned cells, since they have stronger average expression for the immune markers than for any other class of markers. Similarly, we further reassign the epithelial cell as macrophage, since it has strongest expression for the immune markers. After assignment and reassignment, cluster 9 consists of 46 macrophages.

Following the two classification steps, 19 cells remain of undecided cell types, and 58 cells remain unknown. We remove these 77 cells from our dataset, which now consists of 1,112 cells. The resulting distribution of cell types is shown in Table 9.

cell type	epithelial	stroma	macrophage	T cell	B cell	endothelial
number of cells	868	94	64	53	19	14

Supplementary Table 9: Number of single cells per cell type, after classifying cells based on expression markers and clustering, and removing unassigned cells (total: 1,112 cells).

Identifying cycling cells

In identifying the cycling cells, we followed the framework described in (Tirosh et al., 2016), in which scores for the G1/S and G2/M phases of the cell cycle were computed by averaging the expression of a set of relevant genes. Among the 43 genes representative for the G1/S phase, 41 are also identified in our dataset, and among the 55 genes representative for the G2/M phase, we identify 49 in our dataset. *Cycling* cells are defined to be the ones with high G1/S score or G2/M score, and non-cycling cells are the ones with low G1/S and G2/M scores. Unlike the analysis in (Tirosh et al., 2016), which used fixed thresholds for deciding whether a score is high or low, we use data-derived thresholds of 2 MADs above the median, for each score. This amounts to classifying 214 cells as cycling and the remaining 898 as non-cycling.

Identifying copy number alterations from scRNA-seq data

We normalize the expression profiles of our single cells by subtracting, from the expression of each cell, the average expression of 240 normal epithelial cells profiled in a different study (Gao et al., 2017). $20,337$ transcripts are common between our dataset and the dataset profiling the normal epithelial cells. We follow the preprocessing steps for this particular analysis described elsewhere (Tirosh et al., 2016; Patel et al., 2014; Gao et al., 2017), namely we quantify expression as $\log_2(\text{TPM}+1)/10$, and remove all genes with average expression across all cells < 0.1. This amounts to keeping $4,673$ transcripts. We order genes by their chromosomal location, and remove the average expression of the 240 normal cells. We further level all expressions larger than 3 and lower than -3. We define the copy number value of each gene as the sliding average value with a window size of 100 and centered at each gene. Finally, for each gene, we center the resulting copy number values across all cells.

Clustering of epithelial cells

We cluster the 868 epithelial cells in a similar manner as described in the subsection *Clustering*, by using the algorithm developed in `Monocle` and regressing out the patient effect. The number of clusters is automatically chosen by `Monocle`, implementing the density-based approach introduced in (Rodriguez and Laio, 2014). We identify five epithelial clusters, as follows: cluster 1: 22 cells, cluster 2: 398 cells, cluster 3: 231 cells, cluster 4: 170 cells, cluster 5: 47 cells.

Gene expression signatures

We compute the expression of each cell under each of the three **normal breast signatures** (Lim et al., 2009a) by subtracting the mean expression of the downregulated genes from the mean expression of the upregulated genes. We assign each cell to the signature for which it has highest expression. The three normal breast signatures are:

- **mature luminal (ML)**: 561 upregulated genes and 257 downregulated genes, among which 384 upregulated and 197 downregulated genes are also in our data;
- **basal**: 942 upregulated genes and 942 downregulated genes, among which 588 upregulated and 747 downregulated genes are also in our data;
- **luminal progenitor (LP)**: 358 upregulated genes and 179 downregulated genes, among which 231 upregulated and 139 downregulated genes are also in our data.

Similarly, we compute the expression of each cell under each of the four **TNBCtype4 signatures** (Lehmann et al., 2016) by subtracting the mean expression of the downregulated genes from the mean expression of the upregulated genes, and we assign each cell to the signature for which it has highest expression. The TNBCtype4 signatures are:

- **basal like 1**: 320 upregulated genes and 358 downregulated genes, among which 272 upregulated and 291 downregulated genes are also in our data;
- **basal like 2**: 222 upregulated genes and 217 downregulated genes, among which 197 upregulated and 165 downregulated genes are also in our data;
- **mesenchymal**: 314 upregulated genes and 542 downregulated genes, among which 283 upregulated and 448 downregulated genes are also in our data;

- **luminal AR**: 1,093 upregulated genes and 1,143 downregulated genes, among which 923 upregulated and 987 downregulated genes are also in our data.

The **stroma signature** (Lim et al., 2009b) consists of 845 upregulated genes and 396 downregulated genes, among which 501 upregulated and 289 downregulated genes are also in our data. We compute the expression of each cell under this signature by subtracting the mean expression of the downregulated genes from the mean expression of the upregulated genes. The **70-gene prognostic signature** (Van't Veer et al., 2002a,b) consists of 42 genes in our data, the **49-gene metastatic burden signature** (Lawson et al., 2015) consists of 47 genes in our data, and the **354-gene residual tumor signature** (Balko et al., 2012) consists of 281 genes in our data. The **intrinsic basal signature** (Sørlie et al., 2001) consists of 19 genes, among which 17 are also in our data. We compute the expression of each cell under each of these signatures by averaging the mean expression of the genes also found in our data. We plot expression heatmaps using the ComplexHeatmap R package (Gu et al., 2016).

Survival analysis

We perform the survival analysis on the METABRIC mRNA dataset (Pereira et al., 2016), downloaded from the cBioPortal (Gao et al., 2013) on November 20th 2017, and consisting of 299 TNBC patients. We fit Cox proportional hazards regression models, and p-values are obtained from log-rank tests.

Cluster 2 - related signature

We use the function `differentialGeneTest` implemented in `Monocle` to identify genes differentially expressed in cluster 2, as compared to all other epithelial cells. 3,262 genes are differentially expressed at an FDR of 0.1. We evaluate the top 10 differentially expressed genes: *GLTP*, *SKP2*, *HP*, *PGAM5*, *GPI*, *NUDT19*, *INIP*, *ECT2*, *S1PR2*, *MS4A10*, among which 9 (except *MS4A10*) are in the METABRIC dataset. We use as predictor the expression sum of the 9 genes in the signature, and, for visualization purposes, stratify the survival data by quartiles on the number of patients, according to the distribution of the expression sum.

Glycosphigolipid metabolism

The 105 gene members of the glycosphigolipid metabolism pathways (Supplementary Table 4) were obtained by querying The Human Gene Database GeneCards for *sphingolipid metabolism*. Among these, 102 genes (except *STS*, *GM2A*, *GDF1*) are in the METABRIC dataset. We use as predictor the expression sum of the 102 genes in the pathway, and, for visualization purposes, stratify the survival data by tertiles on the number of patients, according to the distribution of the expression sum.

Whole-exome sequencing data

We analyzed exome data from four of the six primary tumors discussed in this study, namely PT039, PT081, PT084, PT089. For normalization purposes, we used the cell line CEPH1408.

Library construction

Prior to library construction, 200 ng (FFPE) or 100 ng (CEPH) of DNA was fragmented to 250 bp and further purified using Agentcourt AMPure XP beads. Size-selected DNA was then ligated to specific adaptors during manual library construction using standard input library prep. Adaptors were chosen following Illumina low-plex guidelines. CEPH1408 required an additional SPRI clean-up to remove high molecular weight DNA following library preparation.

After library construction, all samples were pooled in equal volume and run on a MiSeq nano flowcell to quantitate the amount of library based on the number of observed index reads per sample. All samples yielded sufficient library and were taken forward into hybrid capture. The libraries were pooled at equal mass in two pools to a total of 750 ng each for Exome v5 enrichment using the Agilent SureSelect hybrid capture kit. Normalized captures were then pooled and sequenced on 2 lanes of the HiSeq2500 in Rapid Run mode. The representation of each sample was within the expected range.

Data processing

Pooled samples were demultiplexed using the Picard tools. Read pairs were aligned to the hg19 reference sequence using the Burrows-Wheeler Aligner (Li and Durbin, 2009), and data were sorted and duplicate-marked using Picard tools. The alignments were further refined using the Genome Analysis Toolkit (GATK) (McKenna et al., 2010) for localized realignment around indel sites. Recalibration of quality scores was also performed using the GATK toolbox.

Copy number profiling

Copy number variants are identified using RobustCNV, an algorithm in development at the Center for Cancer Genome Discovery at the Dana-Farber Cancer Institute. RobustCNV relies on localized changes in the mapping depth of sequenced reads in order to identify changes in copy number at the loci sampled during targeted capture. This strategy includes a normalization step in which systematic bias in mapping depth is reduced or removed using robust regression to fit the observed tumor mapping depth against a panel of normals (PON) sampled with the same capture bait set. Observed values are then normalized against predicted values and expressed as log2 ratios. A second normalization step is then done to remove GC bias using a loess fit. Finally, log2 ratios are centered on segments determined to be diploid based on the allele fraction of heterozygous SNPs in the targeted panel.

Normalized coverage data is next segmented using Circular Binary Segmentation (Olshen et al., 2004) with the DNAcopy Bioconductor package. Finally, segments are assigned gain, loss, or normal-copy calls using a cutoff derived from the within-segment standard deviation of post-normalized mapping depths and a tuning parameter which is set based on comparisons to array-CGH calls in separate validation experiments.

We then summarize segment calls to gene calls by assigning segment calls to capture intervals and tallying up interval calls for each gene. Genes may contain multiple intervals with a combination of calls, therefore a variety of gene calls are possible. We use specific rules for determining gene level calls. Since the PON contain samples which have a pattern of systematic bias that is similar to the bias in the tumor samples, this normalization strategy is very effective at removing systematic bias in mapping depth.
